# Engineering non-conventional yeast *Rhodotorula toruloides* for ergothioneine production

**DOI:** 10.1186/s13068-024-02516-2

**Published:** 2024-05-13

**Authors:** Ke Liu, Gedan Xiang, Lekai Li, Tao Liu, Jie Ke, Liangbin Xiong, Dongzhi Wei, Fengqing Wang

**Affiliations:** 1 State Key Laboratory of Bioreactor Engineering, Newworld Institute of Biotechnology, East China University of Science and Technology, Shanghai, 200237 China; 2 Shanghai Key Laboratory of Molecular Imaging, Shanghai University of Medicine and Health Sciences, Shanghai, 201318 China

**Keywords:** Ergothioneine, *Rhodotorula toruloides*, CRISPR-assisted Cre recombination, Metabolic engineering, High-throughput screening

## Abstract

**Background:**

Ergothioneine (EGT) is a distinctive sulfur-containing histidine derivative, which has been recognized as a high-value antioxidant and cytoprotectant, and has a wide range of applications in food, medical, and cosmetic fields. Currently, microbial fermentation is a promising method to produce EGT as its advantages of green environmental protection, mild fermentation condition, and low production cost. However, due to the low-efficiency biosynthetic process in numerous cell factories, it is still a challenge to realize the industrial biopreparation of EGT. The non-conventional yeast *Rhodotorula toruloides* is considered as a potential candidate for EGT production, thanks to its safety for animals and natural ability to synthesize EGT. Nevertheless, its synthesis efficiency of EGT deserves further improvement.

**Results:**

In this study, out of five target wild-type *R. toruloides* strains, *R. toruloides* 2.1389 (RT1389) was found to accumulate the highest EGT production, which could reach 79.0 mg/L at the shake flask level on the 7th day. To achieve iterative genome editing in strain RT1389, CRISPR-assisted Cre recombination (CACR) method was established. Based on it, an EGT-overproducing strain RT1389-2 was constructed by integrating an additional copy of EGT biosynthetic core genes *RtEGT1* and *RtEGT2* into the genome, the EGT titer of which was 1.5-fold increase over RT1389. As the supply of S-adenosylmethionine was identified as a key factor determining EGT production in strain RT1389, subsequently, a series of gene modifications including S-adenosylmethionine rebalancing were integrated into the strain RT1389-2, and the resulting mutants were rapidly screened according to their EGT production titers with a high-throughput screening method based on ergothionase. As a result, an engineered strain named as RT1389-3 was selected with a production titer of 267.4 mg/L EGT after 168 h in a 50 mL modified fermentation medium.

**Conclusions:**

This study characterized the EGT production capacity of these engineered strains, and demonstrated that CACR and high-throughput screening method allowed rapid engineering of *R. toruloides* mutants with improved EGT production. Furthermore, this study provided an engineered RT1389-3 strain with remarkable EGT production performance, which had potential industrial application prospects.

**Supplementary Information:**

The online version contains supplementary material available at 10.1186/s13068-024-02516-2.

## Background

Ergothioneine (EGT) (2-mercaptohistidine trimethylbetaine), a natural chiral thiohistidine derivative, has the function of scavenging reactive oxygen species (ROS), chelating metal ions and protecting cell physiological process [1–4]. EGT can be found universally in high organisms, such as mammals and plants [4, 5]. However, no higher eukaryotes have been reported to biosynthesize EGT. For humans, the human body can only absorb EGT from dietary sources, such as beans, garlic, mushrooms, and meat products, through its own ergothioneine transporter OCTN1, which is responsible for the enrichment of EGT in specific human tissues and cells, such as kidney, lung, and erythrocytes [6, 7]. The EGT level in human body is considered as an important indicator to predict neurodegenerative and cardiovascular disease [8, 9]. Furthermore, EGT is proposed as one of putative vitamins for delaying aging and maintaining health [10]. Therefore, EGT is considered as a safe, valuable dietary supplement, whose therapeutic potential looks promising. Nowadays, a series of EGT-derived nutritional supplements have started to appear, such as ErgoActive, ERGO + and L-ergothioneine. Moreover, numerous well-known EGT-derived cosmetics have also been launched, such as Estee Lauder, ORIGINS, and PROYA.

**Fig. 1:**
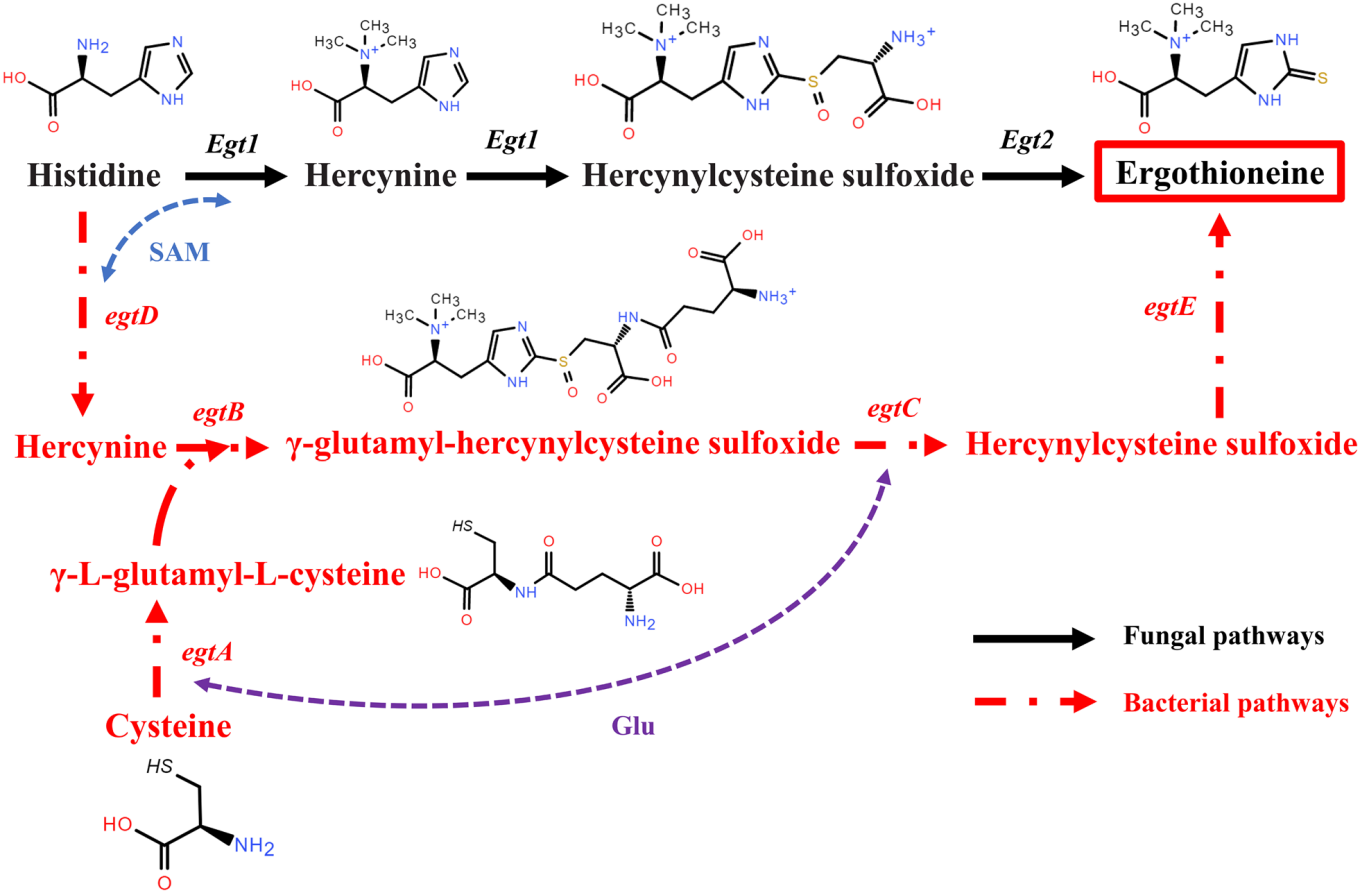
Overview of the EGT biosynthetic pathways in bacteria and fungi

Currently, the preparation routes of EGT mainly include natural extraction [11], chemical synthesis [12], enzymatic reaction [13], and microbial fermentation [4]. Among them, microbial fermentation method has the advantages of mild reaction condition, lower production cost and better sustainable development. Thus, the preparation of EGT by microbial fermentation is a profound research direction. In nature, EGT can be synthesized by numerous organisms, including actinobacteria, filamentous fungi, yeast, cyanobacteria, basidiomycetes, and so on [4, 5, 14–16]. In terms of the different EGT biosynthetic pathways clearly elucidated in these organisms, it is usually classified into bacterial pathway and fungal pathway (Fig. 1). First, both pathways use S-adenosylmethionine (SAM) to transfer three methyl groups to histidine (His) using histidine-specific methyltransferase encoded by *egtD* gene or SAM-dependent methyltransferase encoded by *EGT1* gene, generating hercynine [17, 18]. Next, in the bacterial pathway, γ-L-glutamyl-L-cysteine, synthesized by the condensation of cysteine (Cys) and glutamic acid (Glu) using glutamate cysteine ligase encoded by *egtA* gene, reacts with hercynine using Fe^2+^-dependent oxidase encoded by *egtB* gene, generating γ-glutamyl-hercynylcysteine sulfoxide [19]. Subsequently, the resulting product removes L-glutamate under the catalysis of amidotransferase encoded by *egtC* gene, generating hercynylcysteine sulfoxide (HCO) [20]. However, in the fungal pathway, HCO is directly generated from hercynine using a single EGT1 enzyme [18]. Finally, HCO removes ammonium pyruvate using pyridoxal phosphate (PLP)-dependent C-S lyase encoded by *egtE* gene or PLP-dependent cysteine desulfurase encoded by *EGT2* gene [21, 22], generating our target product, EGT. Compared to the bacterial pathway, fungal pathway has two significant advantages. First, some studies have indicated that NcEGT1 enzyme of *Neurospora crassa* uses Cys instead of γ-L-glutamyl-L-cysteine as a substrate, which eliminates the competing reaction between glutathione and EGT biosynthesis. Second, fungal pathway only involves two enzymes, which are encoded by *EGT1* and *EGT2* genes, respectively [4]. Thus, the EGT biosynthesis pathway of fungi is much simpler than that of bacteria.

To date, with the rapid development of synthetic biology toolkits, several microorganisms have been reconstructed for the biosynthesis of EGT. Among them, the representative model microorganism is *Escherichia coli*. In 2018, Osawa et al. developed a recombinant *E. coli* by heterologous expressing *egtBCDE* genes from *Mycolicibacterium smegmatis*, which was capable of producing 24 mg/L EGT with the addition of His, methionine (Met), thiosulfate as substrate supply [23]. In 2022, Chen et al. reconstituted a novel EGT production system in the *E. coli* by co-expression of two EGT biosynthesis genes (*tregt*1 and *tregt*2) from *Trichoderma reesei*, which could produce 4.34 g/L EGT after 143 h of cultivation in a 2 L jar fermenter [24]. In another study, Zhang et al. reported that the highest EGT production in engineered *E. coli* strain was 5.4 g/L after 94 h in a fed-batch cultivation with 5 L jar fermenter, which was achieved by overexpressing truncated *EGT1* gene from *N. crassa* and *egtD*, *egtE* genes form *M. smegmatis* with multiple rounds of mutations [25]. As for another representative model microorganism, *Saccharomyces cerevisiae*, it was also used to produce EGT. In 2019, Van der Hoek et al. described the engineering of *S. cerevisiae* to produce 598 mg/L EGT in fed-batch culture with 1 L bioreactors by the co-expression of *EGT1* from *N. crassa* and *EGT2* from *Claviceps purpurea*, regulation of the nitrogen metabolism by deleting *TOR1* or *YIH1* genes and optimization of the medium composition [26]. In a follow-up study, Van der Hoek et al. reported that the higher EGT production in engineered *S. cerevisiae* strain was 2.39 g/L after 160 h in fed-batch fermentation with 1 L Sartorius bioreactors, which was achieved by the directed mutagenesis screening of His overproducing mutant based on His analog β-(1,2,4,-triazol-3-yl)-DL-alanine (TRA), integration *MET14* gene, deletion of *SPE2* gene and medium optimization [27].

As an attractive alternative, the non-conventional yeast *Rhodotorula toruloides* has emerged as a promising platform organism for its excellent bioengineering potential and microbial safety. Specifically, *R. toruloides* has the ability to utilize a broad spectrum of substrates, including saccharides, alcohols, organic acids, biomass hydrolysates, some industrial wastewaters, and so on. Additionally, it has a well-developed mevalonic acid (MVA) pathway, which contributes to naturally accumulating numerous basic precursor substances and bulk chemicals, such as isoprene, acetyl-CoA, carotenoids, and lipids [28, 29]. Not only that, it has some beneficial characteristics, such as acid-resisting, high temperature-resisting, high fermentation density, and strong robustness. More importantly, feed supplemented with a *Rhodotorula* cell mass has been found to be safe and non-toxic in animals [28–30]. Therefore, *R. toruloides* is an efficient, green, and economical natural cell factory, which is conducive to large-scale industrial production. Furthermore, the possible EGT synthetic gene cluster, composed of *RtEGT1* (RHTO_05467) and *RtEGT2* (RHTO_04309), is annotated in *R. toruloides* NP11 (GenBank: GCA_000320785.2) [31], which indicates that this strain may be able to synthesize EGT. These advantages prompt us to perform genetic engineering of *R. toruloides* for overproduction of the desired products, specifically EGT.

In the present study, we aimed to engineer *R. toruloides* to obtain a potential host for industrial EGT production. For this purpose, we characterized the corresponding EGT production of five wild-type *R. toruloides* to find the chassis strain, *R. toruloides* 2.1389, for subsequent genetic engineering. Next, to handle the problem of iterative genome editing in *R. toruloides*, we developed a simple gene editing method for gene knockout and random integration by the combined utilization of CRISPR-Cas9 and Cre-loxp system. Furthermore, we established a high-throughput method which could rapidly assess the EGT concentration in 96-well plate culture. As a result, we engineered a *R. toruloides* mutant RT1389-3 with hyper-productivity of EGT by integration of endogenous EGT synthetic gene cluster and SAM rebalancing-related genes, which could produce 267.4 mg/L EGT at the shake flask level. Our work provided an efficient genome editing and high-throughput screening method, and resultful engineering strategy for improving EGT production, which might benefit efficient biosynthesis of other higher-value products.

## Results and discussion

### Screening of the parental strain of R. toruloides for EGT production

**Fig. 2:**
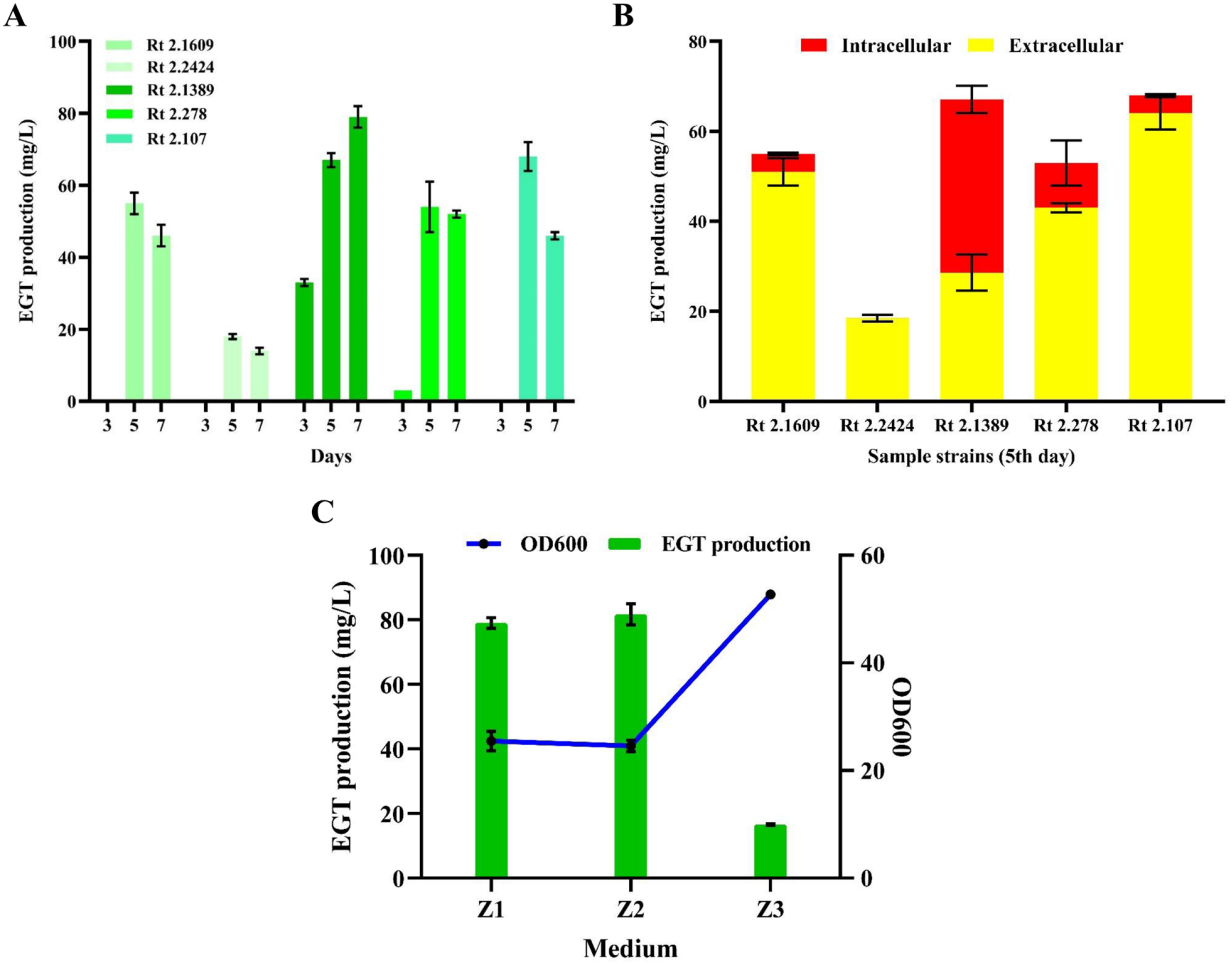
EGT production performance of wild-type *R. toruloides* in Z1 medium.** A** EGT production of strains Rt 2.1609, Rt 2.2424, Rt 2.1389, Rt 2.278 and Rt 2.107 on days 3, 5, and 7. *Rt R. toruloides*. **B** The intracellular and extracellular EGT production of strains Rt 2.1609, Rt 2.2424, Rt 2.1389, Rt 2.278 and Rt 2.107 on day 5. **C** Growth condition and EGT production of Rt 2.1389 in Z1, Z2, and Z3 media on day 7

Obviously, different types of *R. toruloides* have various abilities to biosynthesize EGT due to their own physiological conditions. A good chassis strain is more conducive to achieving our objectives. Thus, in the present study, five different *R. toruloides* strains, including *R. toruloides* 2.1609, *R. toruloides* 2.2424, *R. toruloides* 2.1389 (RT1389), *R. toruloides* 2.278, and *R. toruloides* 2.107 (Table 1), were chosen to examine their respective EGT production with 50 mL original Z1 fermentation medium at 220 rpm for 7 days. As expected, these *R. toruloides* strains could independently synthesize EGT, the total titer of which ranged from 14.6 mg/L to 79.0 mg/L (7th day) (Fig. 2A, Figure S1). Among them, the highest EGT titer was accumulated in RT1389. Additionally, of the five *R. toruloides* strains, four accumulated EGT both inside and outside the cell, except *R. toruloides* 2.2424 (5th day) (Fig. 2B). While the fermentation process lasted to the 7th day, all strains mainly accumulated EGT outside the cell. Together, the result indicated that RT1389 could serve as a potential platform for EGT production. On the one hand, it naturally synthesized EGT due to its innate EGT biosynthesis genes. On the other hand, it mainly produced EGT outside the cell with the fermentation prolonging to the 7th day, which was helpful in the subsequent separation and purification of EGT. Therefore, the strain RT1389 was selected for the following construction of EGT overproduction strains.

**Table 1:** Strains used in this study

Names	Descriptions	Sources
*Escherichia coli* DH5α	General plasmid cloning host	Transgen biotech
*Agrobacterium tumefaciens GV3101*	*Agrobacterium tumefaciens*, *Gen*^*R*^, *Rif*^*R*^	Shanghai zeye biotechnology
*R. toruloides* 2.1609	*R. toruloides* wide-type strain	CGMCC
*R. toruloides* 2.2424	*R. toruloides* wide-type strain	CGMCC
*R. toruloides* 2.1389	*R. toruloides* wide-type strain	CGMCC
*R. toruloides* 2.278	*R. toruloides* wide-type strain	CGMCC
*R. toruloides* 2.107	*R. toruloides* wide-type strain	CGMCC
RT1389-1	Integration of effective CRISPR-SpCas9 and Cre-loxp system in *R. toruloides* 2.1389	This study
E1-E2	Random integration of RtEGT1-RtEGT2 dual expression cassette in RT1389-1	This study
E1-p1-E2	Random integration of p2A1 sequence mediated RtEGT1-RtEGT2 co-expression cassette in RT1389-1	This study
E1-p2-E2	Random integration of p2A2 sequence mediated RtEGT1-RtEGT2 co-expression cassette in RT1389-1	This study
RT1389-2	A type of *R. toruloides* strain with highest EGT production among E1-E2, E1-p1-E2 and E1-p2-E2 strains	This study
Met14	Random integration of Met14 expression cassette in RT1389-1	This study
SAM2	Random integration of SAM2 expression cassette in RT1389-1	This study
SAH1	Random integration of SAH1 expression cassette in RT1389-1	This study
ADO1	Random integration of ADO1 expression cassette in RT1389-1	This study
△SPE2	Deletion of *SPE2* gene in RT1389-1	This study
ADO2	Random integration of ADO1 expression cassette in RT1389-2	This study
SAM2-SAH1	Random integration of p2A1 sequence mediated SAM2-SAH1 co-expression cassette in RT1389-2	This study
Met14-SAM2-SAH1	Random integration of p2A1 sequence mediated Met14-SAM2-SAH1 co-expression cassette in RT1389-2	This study
RT1389-3	A type of *R. toruloides* strain with highest EGT production among ADO2, SAM2-SAH1 and Met14-SAM2-SAH1 strains	This study

Subsequently, EGT production and growth condition of strain RT1389 were further characterized in other Z2 and Z3 fermentation media. The results showed that there was no significant difference in EGT production of RT1389 when using Z1 and Z2 media (7th day) (Fig. 2C). However, for Z3 medium, the EGT production was only 16.6 mg/L, which was significantly lower than that of Z1 and Z2 media, while the growth condition of RT1389 was clearly superior to that of the other medium. These results indicated that the C/N ratio might have a critical impact on the EGT production of RT1389. Considering the relatively simple components and highest EGT yield of Z2 medium, it was chosen for subsequent experiments. To further understand the performance of strain RT1389 in biosynthesizing EGT in Z2 medium, its growth condition and EGT production were measured at the shake flask level. The results showed that the growth rate of RT1389 did not keep pace with the productive rate of EGT (Figure S2A, B). Thereinto, 0–12 h was the lag phase, and 12–66 h was the logarithmic phase. And then, within the following 3–11 days, the OD_600_ value of RT1389 gradually decreased. However, the EGT production was very low in the first three days, and gradually increased until reaching its highest level on the 7th day, and then started to decrease. Thus, for RT1389, the flask fermentation time was mainly controlled on the 7th day. Additionally, it was worth noting that there was a significant decrease in EGT production during the later stage of fermentation, indicating that this strain might have a potential EGT degradation mechanism.

### Supplementation of precursor amino acids for improving EGT production

**Fig. 3:**
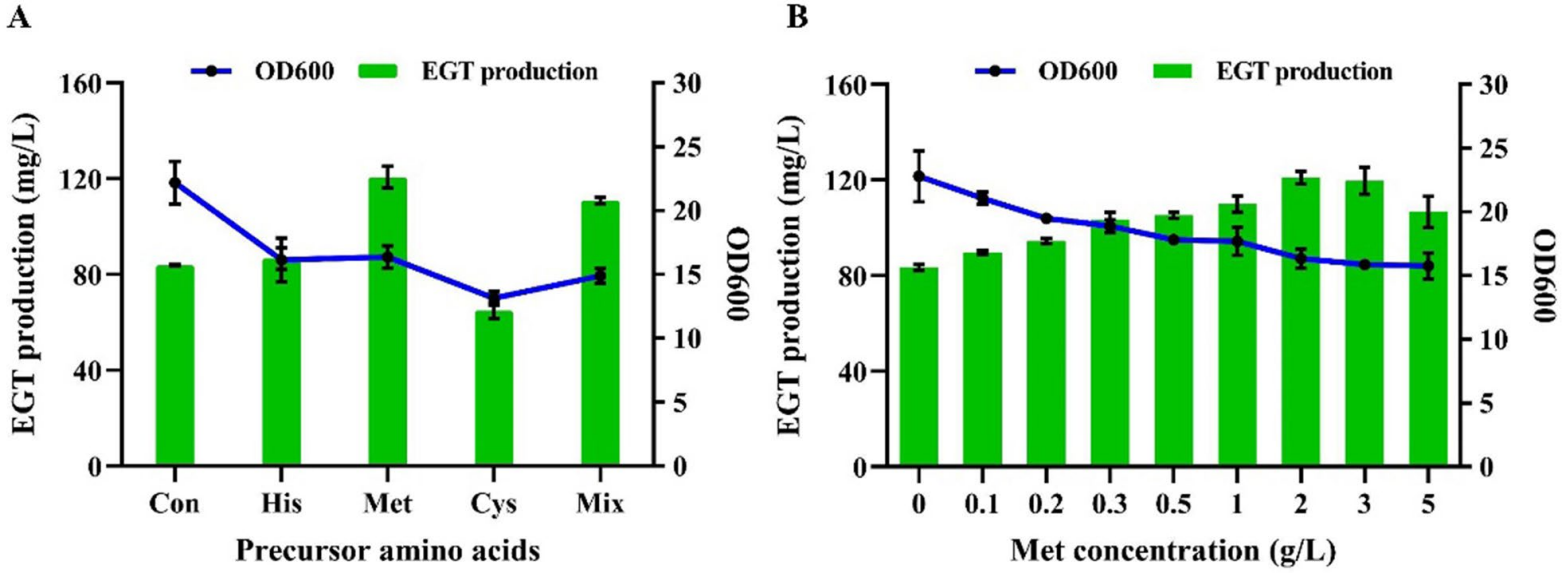
Effect of supplementation of precursor amino acids on EGT production of RT1389 strain in Z2 medium. **A** Effect of supplementation of Met, Cys, His, Mix on EGT production. *Con* Control group. *Mix* mixture of three amino acids. **B** Effect of supplementation of different concentrations of Met on EGT production

The biosynthesis process of EGT mainly involves three precursor amino acids, among which His provides the skeleton, Cys provides the thiol group, and Met provides the methyl group [27]. To determine whether the supply of precursor amino acids is conducive to accumulating EGT, the strain RT1389 was cultivated in 50 mL original Z2 fermentation medium with supplementation of 3 g/L of each of Met, Cys, His, or 3 g/L mixture of three amino acids, respectively. As shown in Fig. 3A, the additional supplementation of Met improved EGT levels up to 43.9% compared to the original Z2 medium, while the supplementation of His did not significantly improve EGT production and the supplementation of Cys, however, reduced EGT levels by 22.6%. Considering that Met is mainly converted into SAM through SAM cycle, and then is incorporated into the synthetic route of EGT (Fig. 1, Fig. 5B), it was speculated that insufficient supply of Met hindered the acquisition of the skeleton derived from His, which led to limited production capacity of hercynine. That is, the lack of Met was the main factor for limiting the synthesis efficiency of EGT in strain RT1389. Similarly, due to the insufficient hercynine production, high concentrations of Cys could not be duly converted into HCO, which had a negative impact on the growth conditions of strain RT1389 and led to a decrease in EGT production.

Therefore, further research was conducted to investigate the effects of different concentrations of Met supplementation on EGT production and growth condition of RT1389. As shown in Fig. 3B, when additive amount of Met was 2 g/L, the EGT production was the highest, reaching 120.9 mg/L. Furthermore, it was noticed that the growth condition of RT1389 gradually deteriorated and the EGT production showed a trend of first rising then descending with the continuous increase in concentrations of Met supplementation. Clearly, too-high or too-low concentrations of Met were not conducive to the accumulation of EGT. Based on the original Z2 fermentation medium supplemented with 2 g/L Met, the effects of low concentration of Cys supplementation on RT1389 were further investigated at the shake flask level. As shown in Fig. S3, low concentration of Cys supplementation (0–0.6 g/L) could promote EGT accumulation, while high concentration of Cys supplementation (> 0.6 g/L) had the opposite effect. When the addition amount of Cys was 0.6 g/L, the strain had the highest EGT production of 158.5 mg/L. Nevertheless, Cys was difficult to dissolve in water, and its aqueous solution was unstable, which was not conducive to the preparation of fermentation medium. Thus, Z2 medium with only Met supplementation in subsequent experiments remained the preferred choice.

### CACR-mediated iterative gene manipulation

**Fig. 4:**
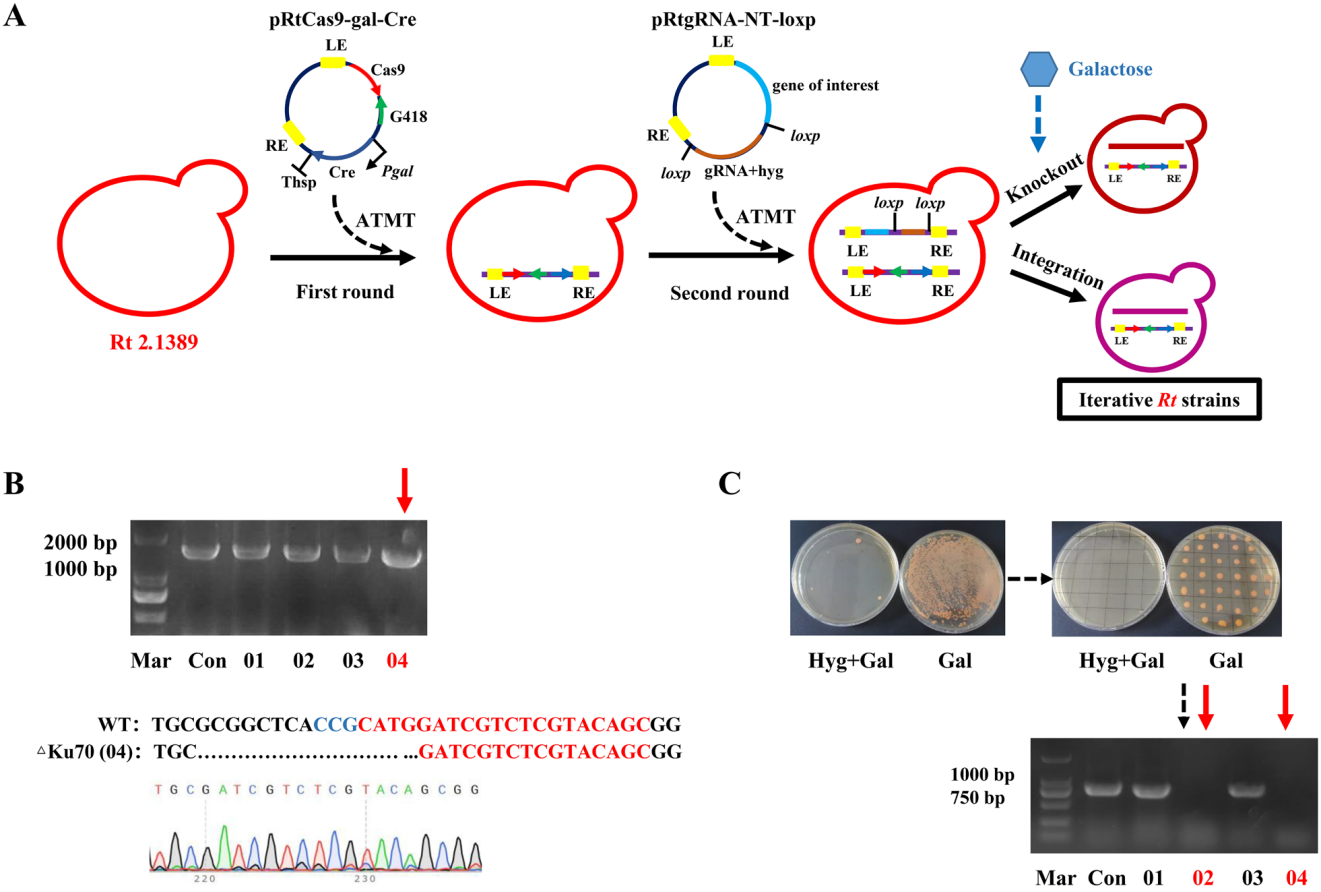
CACR-mediated iterative genetic manipulation of RT1389 strain. **A** The schematic diagram of gene knockout and integration mediated by CACR method. *LE* Left border repeat from nopaline C58 T-DNA. *RE* Right border repeat from nopaline C58 T-DNA. *G418* geneticin-resistant fragment. **B** The results of *Ku70* gene knockout mediated by CACR method. *Mar* DNA ladder marker. The red arrow points to the correct sample with *Ku70* gene knockout. 01–04 denotes four different strain samples 01–04 grown on the screening plate. **C** The results of *Hyg* resistance gene deletion mediated by CACR method. After adding galactose, the galactose induced promoter *Pgal1* could induce the expression of Cre recombinase to accurately excise the *Hyg* resistance fragment between two loxp sites, and then the resultful positive transformants were unable to grow on the plate containing hygromycin antibiotic. *Hyg* + *Gal* Hygromycin antibiotic and galactose inducer. The red arrow points to the correct sample with *Hyg* gene excision

Recent progress on synthetic biology toolkits of *R. toruloides*, including endogenous expressing elements [32, 33], site-specific recombinase systems [34], and CRISPR-based genome editing tools [35–37], enable us to perform efficient genetic manipulation of *R. toruloides* genome. However, due to the lack of endogenous free plasmids and any chromosomal autonomous replication sequences or centromere sequences that can serve as replication elements in *R. toruloides*, it remains a challenge to implement iterative gene editing [29]. Iterative gene editing is one of the fundamental biotechnologies in the development of microbial cell factories for large-scale industrial applications. Thus, to accomplish iterative genetic manipulation of strain RT1389, the application of CRISPR-assisted Cre recombination (CACR) method was described in this study (Fig. 4A). Specifically, in the first round, the plasmid pRtCas9-gal-Cre, carrying SpCas9 and inducible Cre expression cassette, was integrated into RT1389 through *Agrobacterium tumefaciens*-mediated transformation (ATMT) method, generating the transformants RT1389-Cre/Cas9-G418. In the second round, a series of pRtgRNA-NT-loxp-derived plasmids, carrying gRNAs or genes of interest, were introduced into the strain RT1389-Cre/Cas9-G418, generating the expected positive transformants. In the third round, for the next round of iterative gene editing, the *Hyg* resistance gene and the superfluous integrated fragment from the genome of positive transformants in the second round were accurately deleted by inducing the expression of Cre recombinase. Based on this principle, CACR system could achieve iterative gene mutation and random gene integration (Fig. 4A).

Considering that ATMT method involves integrating transfer DNA (T-DNA) into *R. toruloides* genome via random insertion [38, 39], a transformant Rt1389-Cre/Cas9-G418, which could perform normal CRISPR-SpCas9 and Cre-loxp system functions, should be selected as the chassis strain for our subsequent research. In this study, *Ku70* gene (RHTO_06014, encodes a Ku70 protein involved in the non-homologous end joining (NHEJ) pathway) was chosen as the test subject to screen a viable transformant Rt1389-Cre/Cas9-G418 [31]. Specifically, in the first round, the plasmid pRtgRNA-Ku70-2-loxp was introduced into four independent Rt1389-Cre/Cas9-G418 transformants, respectively, and then the resulting transformants were detected through Sanger sequencing using the primers pairs Ku70-F/Ku70-R. As a result, only the strain 04 could effectively mediate *Ku70* gene disruption (Fig. 4B, Table S3). Therefore, in the second round, the strain 04 was induced to express Cre recombinase by adding inducer galactose, the resulting transformants of which were also verified by resistance plate screening test and PCR identification using the primer pairs Hyg-F/Hyg-R. The results indicated that 2 transformants (50%) out of 4 accomplished *Hyg* resistance gene deletion (Fig. 4C). Together, the transformant Rt1389-Cre/Cas9-G418 corresponding to strain 04 was confirmed to perform CRISPR-SpCas9 and Cre-loxp system functions normally, and was renamed as RT1389-1 for ease of differentiation (Table 1).

To further evaluate the performance of strain RT1389-1, on the one hand, the *PAL* gene (RHTO_01960) was selected as the test subject due to its encoding of phenylalanine ammonia-lyase, which might be related to the degradation of EGT [4]. Specifically, the plasmids pRtgRNA-PAL-1-loxp, pRtgRNA-PAL-2-loxp, and pRtgRNA-2PAL-loxp, carrying sgRNA-PAL1, sgRNA-PAL2, and sgRNA-2PAL expression cassette targeting *PAL* gene (Table S3), were introduced into strain RT1389-1, respectively, and then the resulting transformants were detected through Sanger sequencing using the primers PAL-cx-F/PAL-cx-R. As expected, relying on the endogenous NHEJ pathway, the CACR system in strain RT1389-1 could mediate single and dual target gene editing, and DNA sequencing results showed that target DNA sequences exhibited diverse mutation types, including indefinite number of base deletions and single base insertion mutation (Figure S4, Table S3). On the other hand, the EGT production and the growth condition of strain RT1389-1 were further characterized in Z2 fermentation medium. The results showed that there were no obvious changes in the growth and EGT production of strains RT1389 and RT1389-1 (Figure S5). Collectively, these results indicated that integration of CACR system in the strain RT1389-1 allowed for iterative gene editing and further metabolic engineering to produce EGT.

### Overexpression of EGT biosynthetic genes and regulation of SAM pathway genes

**Fig. 5:**
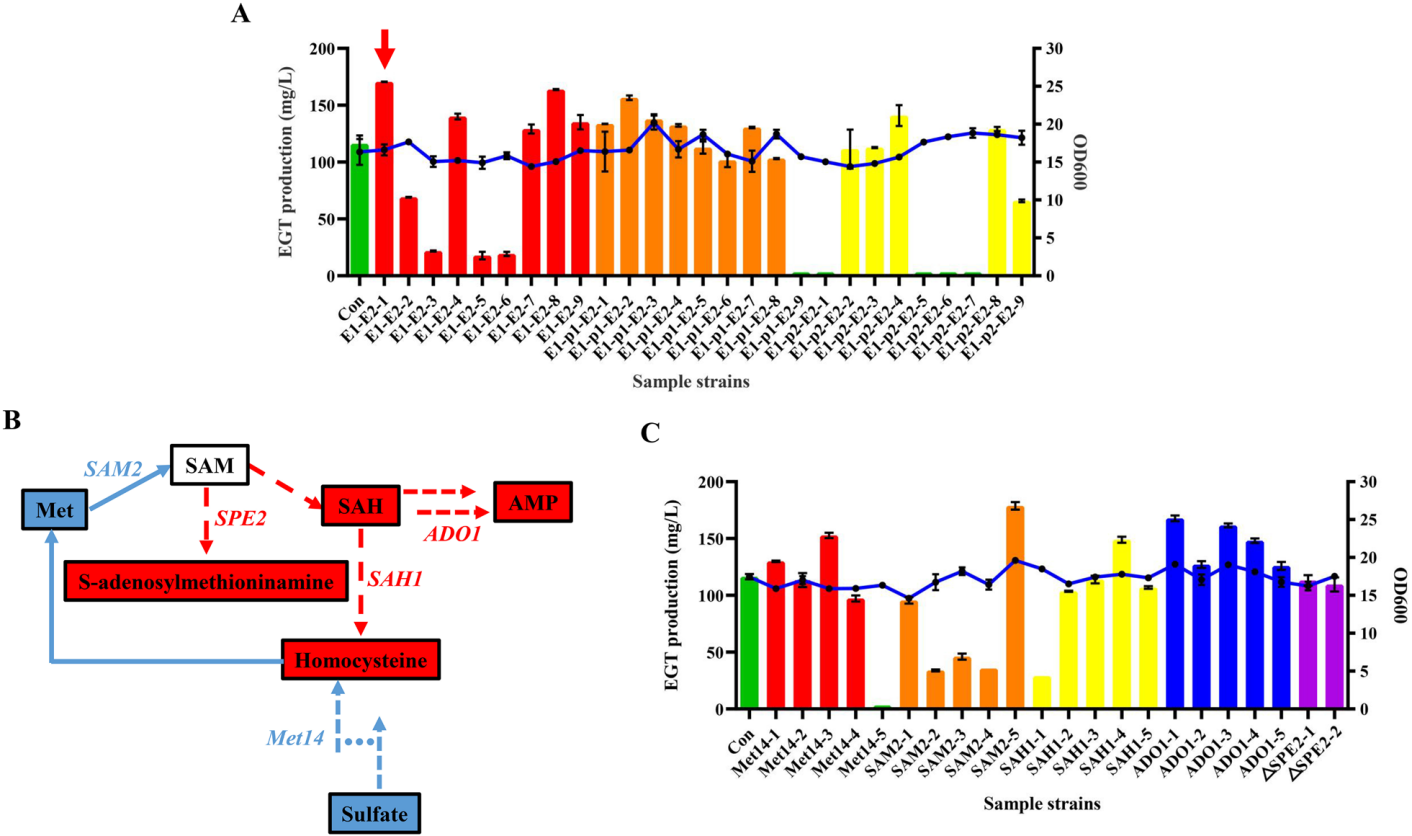
Effect of overexpression of EGT biosynthetic genes or regulation of SAM pathway genes via ATMT process on EGT production of RT1389-1 strain in modified Z2 medium. **A** Effect of overexpression of *RtEGT1* and *RtEGT2* genes on EGT production. *E1*-*E2* transformants with additional RtEGT1-RtEGT2 dual expression cassette. *E1*-*p1-E2* transformants with additional p2A1 sequence mediated RtEGT1-RtEGT2 co-expression cassette. *E1*-*p2-E2* transformants with additional p2A2 sequence mediated RtEGT1-RtEGT2 co-expression cassette. Column and broken line denote EGT production and OD_600_, respectively. 1–9 denotes randomly selected samples 1–9. The red arrow points to the sample with highest EGT production. **B** A brief schematic diagram of the SAM cycle. *SAH* S-adenosylhomocysteine. *AMP* Adenosine monophosphate. **C** Effect of overexpression of *Met14*, *SAM2*, *SAH1*, *ADO1* genes or deletion of *SPE2* gene on EGT production, respectively

The most direct method for improving EGT production is overexpression of the enzymes related to EGT biosynthesis [4]. Therefore, to further enhance the synthetic capacity of EGT of strain RT1389-1, three types of plasmids, pRtEGT1-EGT2, pRtEGT1-p2A1-EGT2 and pRtEGT1-p2A2-EGT2, carrying RtEGT1-RtEGT2 dual expression cassette, *Porcine teschovirus*-1 2A (p2A1)-mediated RtEGT1-RtEGT2 co-expression cassette, and *Zaire ebolavirus* 2A (p2A2)-mediated RtEGT1-RtEGT2 co-expression cassette [40], were introduced into RT1389-1 through ATMT method, respectively. Considering that ATMT-mediated genomic integration is random and different genomic integration sites may have an impact on the biosynthesis of EGT, 9 corresponding positive samples from the above three types of transformants were randomly selected for fermentation testing with modified Z2 medium, respectively. As a result, the EGT production of different transformants varied greatly, which ranged from 0 mg/L to 170.5 mg/L, while the growth of these strains did not change significantly, the OD_600_ value of which ranged from 14.4 to 20.2 (Fig. 5A). This phenomenon was consistent with the previous viewpoint about ATMT-mediated random integration. Furthermore, the reason for some strains with the EGT production of 0 mg/L were investigated whether it was related to unexpected disruption of *RtEGT1* or *RtEGT2* genes. However, DNA sequencing results showed that none of these genes were changed. Also, real-time fluorescence quantitative PCR (qPCR) was performed to measure level of changes of mRNA level (2^−ΔΔCt^) of *RtEGT1* and *RtEGT2* genes in *R. toruloides* engineering strains E1-E2-1 and E1-E2-5, the EGT production of which were 170.5 mg/L and 17.8 mg/L, respectively. The results showed that the transcriptional level of *RtEGT1* and *RtEGT2* genes in strain E1-E2-1 were 77.2-fold and 2.2-fold increase over control strain RT1389-1, while that in strain E1-E2-5 were 2.6-fold and 0.4-fold, respectively (Figure S6). That is, additional expression of *RtEGT1* and *RtEGT2* genes might be conducive to EGT production, while low-level expression of *RtEGT2* gene might have the opposite effect. Additionally, among these randomly selected samples, it was found that p2A1 and p2A2 sequences could mediate RtEGT1-RtEGT2 co-expression for the increase in EGT production. Specifically, 5 E1-p1-E2 transformants (55.6%) out of 9 and 1 E1-p2-E2 transformant (11.1%) out of 9 mediated the upregulation of EGT production, while the positive proportion of E1-E2 transformants was 66.7%, and the strain E1-E2-1 with the highest EGT production derived from E1-E2 transformants (Fig. 5A). This phenomenon was deemed to be closely related to the ATMT process. Subsequently, the *Hyg* resistance gene of strain E1-E2-1 was excised through CACR method, generating the strain RT1389-2.

Meanwhile, considering that the initial steps of EGT biosynthesis involve the transfer of three methyl groups derived from SAM [4], rebalancing the SAM pathway in RT1389-1 cells might also be conducive to accumulating EGT (Fig. 1). Therefore, several SAM pathway-regulatory genes, including *Met14*, *SAM2*, *SAH1*, *ADO1*, and *SPE2* genes (Fig. 5B) [27, 41], were identified by sequence alignment with the homologs from *Saccharomyces cerevisiae* S288C (GenBank: GCA_022626425.1) (Table S4). Whereafter, these corresponding gene overexpression plasmids, including pRtMet14, pRTSAM2, pRTSAH1, pRTADO1, and gene knockout plasmid, pRtgRNA-SPE2-loxp, were introduced into the strain RT1389-2 by ATMT method, respectively. Similarly, for the above transformants, 5 corresponding positive samples were randomly selected for shake flask fermentation testing, respectively. As a result, surprisingly, 5 *ADO1* transformant (100%) out of 5 increased EGT production (Fig. 5C). This largely indicated that the adenosine kinase encoded by *ADO1* gene may help to enhance methyl cycle by increasing the consumption of adenosine, which was conducive to improving EGT production. However, only 4 of the remaining gene transformants showed an increase in EGT production. This also demonstrated that the ATMT process had a significant impact on EGT biosynthesis, making it difficult to directly and accurately evaluate the positive and negative effects of different genes. In comparison, for *SPE2* gene, due to the limited gene knockout efficiency of the CACR system (Figure S4, Table S3), two positive mutant strains were chosen to perform shake flask fermentation testing. However, there was no significant change in the EGT production of two mutants (Fig. 5C). That is, unlike *S. cerevisiae*, deletion of *SPE2* might not promote the accumulation of EGT in *R. toruloides* by preventing the utilization of SAM.

### High-throughput screening of EGT-overproducing engineered strain by enzymatic quantification of ergothionase

**Fig. 6:**
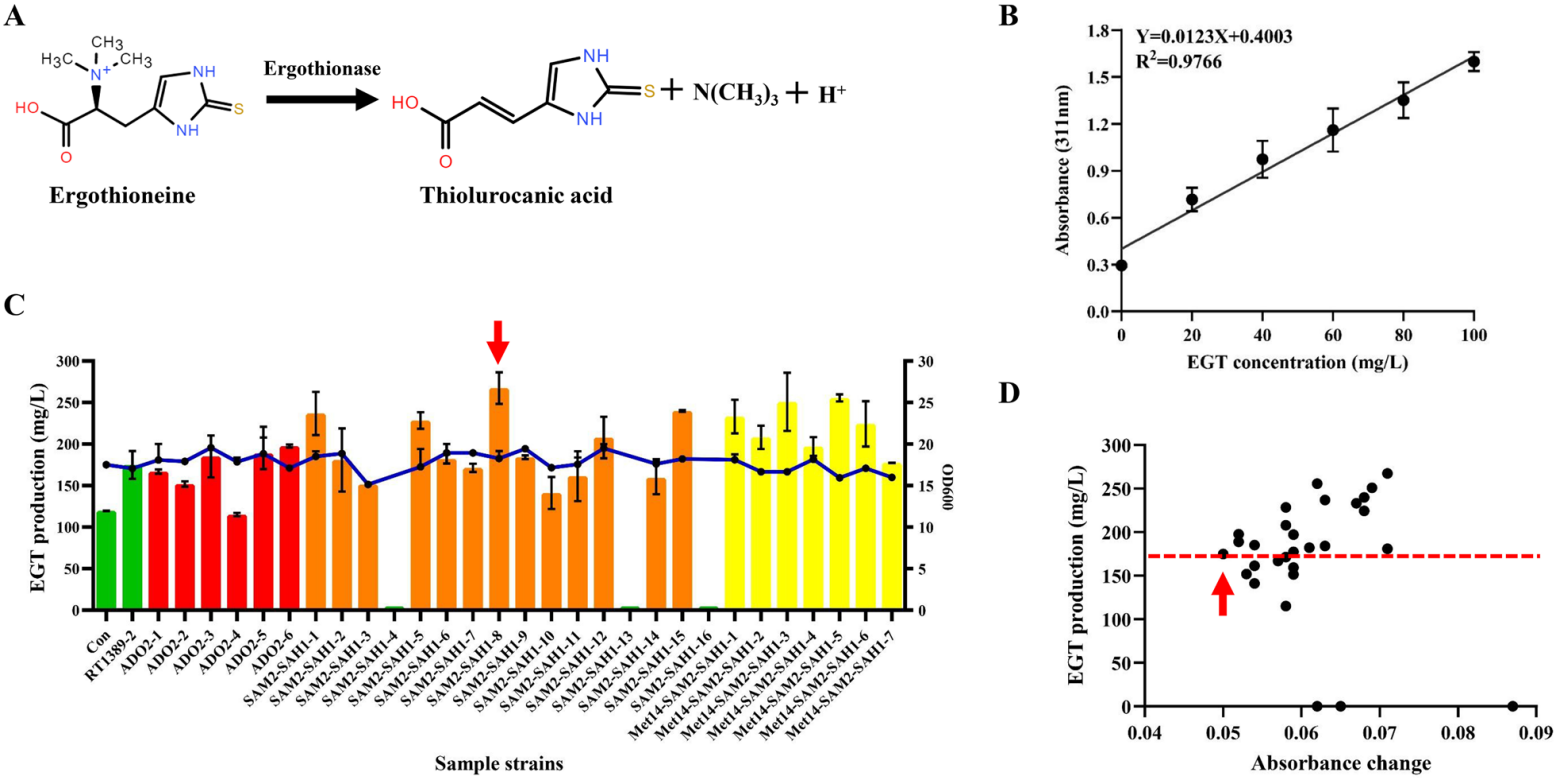
Results of high-throughput screening of EGT-overproducing engineered strain by enzymatic quantification of ergothionase. **A** The schematic diagram of reaction catalyzed by ergothionase. **B** The linear relationship between the concentration of EGT standard and the absorbance detected with a microplate reader at 311 nm at room temperature for 30 min. **C** Effect of overexpression of *ADO1* one gene, *SAM2*-*SAH1* two genes and *Met14*-*SAM2*-*SAH1* three genes via ATMT process on EGT production of RT1389-2 strain in modified Z2 medium. Among them, strains sample SAM2-SAH1-4, SAM2-SAH1-13 and SAM2-SAH1-16 did not grow in shake flask fermentation. The red arrow points to the sample with highest EGT production. **D** The relationship between the concentration of EGT in the fermentation broths and the absorbance change of 29 engineering strains at 311 nm. The red arrow points to the control sample RT1389-2

In previous studies, to handle random integration events of ATMT process, the most common strategy is to integrate target fragment into a specific gene site via homologous recombination (HR) [32]. However, it is difficult to implement targeted gene manipulation due to the extremely low efficiency of HR in *R. toruloides* [29]. In the present study, inversely, the ATMT process was utilized to introduce numerous uncertain genomic mutations for fast stimulation of the EGT production performance of *R. toruloides*. To achieve this purpose, a simple high-throughput analysis method of EGT concentration was established in this study based on ergothionase (Figure S7). The basic principle of this method is that ergothionase from *Burkholderia* sp. HME13 can effectively catalyze EGT to produce thiol urocanic acid, which can be detected at 311 nm on a microplate reader (Fig. 6A) [25, 42]. To test the feasibility of this principle, 10 μL of ergothionase crude extract was added to 190 μL of 0 − 100 mg/L EGT standard at 37 ℃ for 30 min, and then the absorbance of reaction mixture was detected with a microplate reader at 311 nm. The results showed that the concentration of EGT (0–100 mg/L) was approximately proportional to the absorbance value (0.294–1.598) (Fig. 6B), indicating that the ergothionase from *Burkholderia* sp. HME13 was suitable for rapid assessment of EGT concentration.

Based on the above analysis strategy, to obtain a mutant with higher EGT yield, the effects of *Met14*-*SAM2*-*SAH1* three genes, *SAM2*-*SAH1* two genes, and *ADO1* one gene on EGT production were further characterized based on the platform strain RT1389-2. To simplify plasmid construction, the previously well performing p2A1 sequence was used to mediate co-expression of these genes. Specifically, three types of plasmids, pRtSAM2-p2A1-SAH1-p2A1-Met14, pRtSAM2-p2A1-SAH1, and pRtADO1, carrying SAM2-SAH1-Met14, SAM2-SAH1, and ADO1 expression cassette, were introduced into RT1389-2 strain through ATMT method, respectively. The resulting 192 transformants were inoculated into 96-well plates with 600 μL modified Z2 medium, and were rapidly analyzed the absorbance change (see methods). Due to the absorbance change of strains RT1389-1 and RT1389-2 being approximately 0.03 and 0.05, respectively, only strains with absorbance change greater than 0.05 were selected for further shaking flask fermentation testing. The results showed that 6 ADO2 strains (6.3%) out of 96, 16 SAM2-SAH1 strains (22.2%) out of 72 and 7 Met14-SAM2-SAH1 strains (29.2%) out of 24 mediated a change in absorbance value greater than 0.05 (Figure S8). Contrary to previous observations of ADO1 strains, for ADO2 strains, it could be inferred that the randomness of the ATMT-mediated *ADO1* gene integration process had a more important impact on the EGT yield of RT1389-2 strain. Moreover, SAM2-SAH1 strains and Met14-SAM2-SAH1 strains also presented similar results.

Subsequently, 29 engineering strains mentioned above were selected for further shaking flask fermentation testing. Among them, 18 strains (62.1%) out of 29 mediated the upregulation of EGT production, which ranged from 1.4% to 52.8% over RT1389-2 strain (Fig. 6C), and the highest EGT production reached 267.4 mg/L. Overall, it largely demonstrated the feasibility of the aforementioned high-throughput analysis method. Though there was no strict linear relationship between absorbance change and EGT production, however, it could be roughly seen that the greater absorbance change, the higher probability of high EGT production (Fig. 6D, Figure S8). Furthermore, the corresponding strain with highest EGT production was easily excised *Hyg* resistance gene, generating the finally engineering strain RT1389-3.

### Shaking flask fermentation analysis

**Fig. 7:**
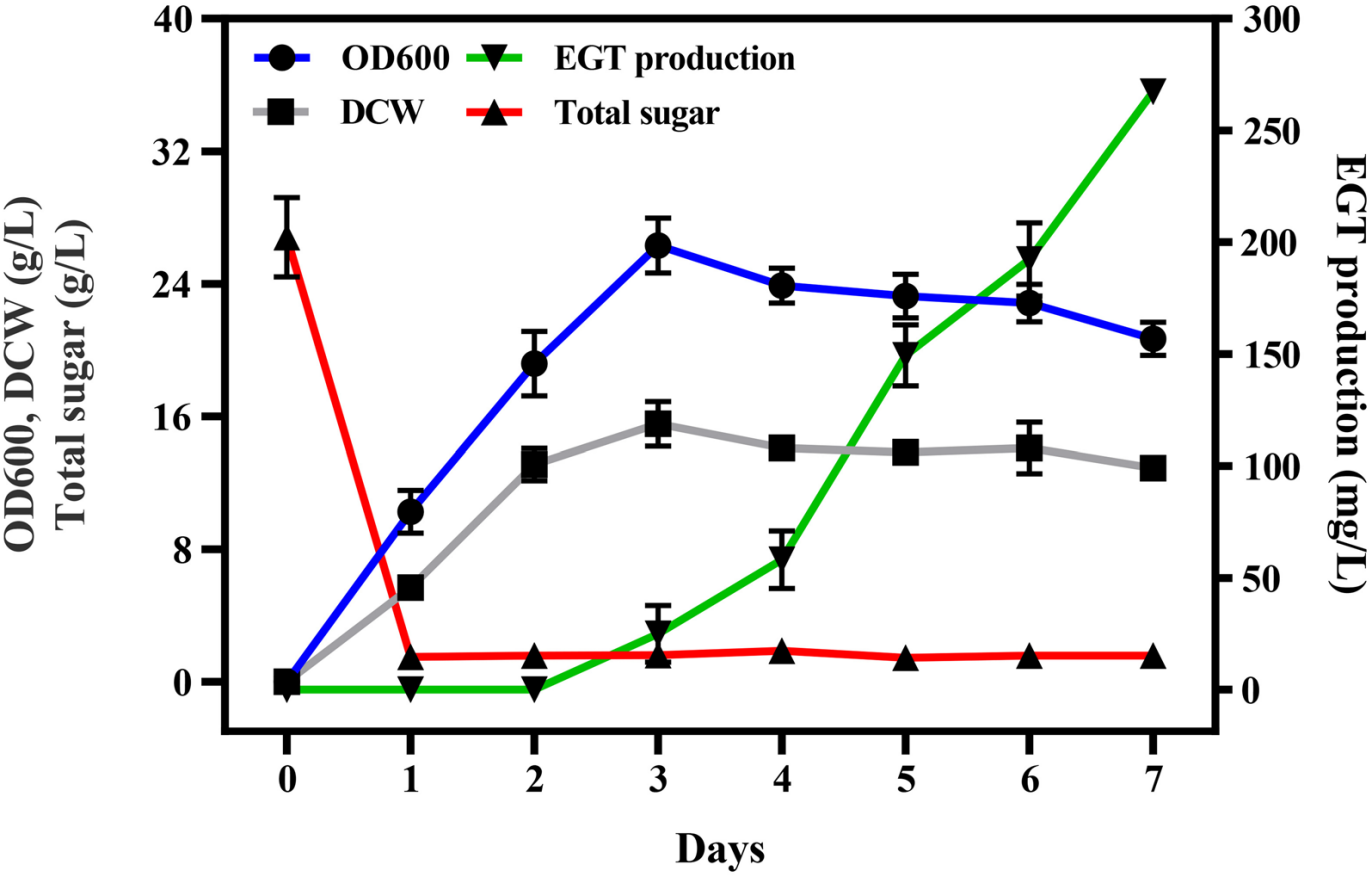
Shaking flask fermentation analysis of RT1389-3 with modified Z2 medium. *DCW* dry cell weight

Finally, the strain RT1389-3 was cultivated in a 50 mL modified Z2 medium for fermentation analysis of EGT. During the fermentation process, the cell density and the biomass of RT1389-3 increased first and then decreased slowly. After 7 days of fermentation, RT1389-3 produced 267.4 ± 4.4 mg/L EGT with dry cell weight (DCW) of 12.9 ± 0.5 g/L, which was equal to a productivity of 1.6 mg/L/h and a product yield of 10.6 mg EGT/g glucose (Fig. 7). Approximately 96% of the produced EGT was retained extracellularly. Compared to other similar *Rhodotorula* species used for EGT production [43, 44], our strain showed a 300% increase from the reported EGT productivity at 0.4 mg/L/h in *R. mucilaginosa* DL-X01 with the same volume of fermentation broth (titer of 38.5 ± 2.7 mg/L in 96 h), and could also be further iterable engineered for better EGT production performance with CACR and high-throughput screening technologies developed in this study.

Meanwhile, given that the potential of *R. toruloides* for converting underutilized and abundant carbon sources, such as xylose, into high-value products [45], RT1389-3 was also cultivated in a 50 mL modified Z2 medium containing xylose as a carbon source for fermentation analysis of EGT. Xylose concentration was set at 25 g/L. During the fermentation process, the cell density of RT1389-3 increased continuously. After 7 days, RT1389-3 produced 42.6 ± 3.8 mg/L EGT (Figure S9). The results demonstrated that RT1389-3 could assimilate xylose even without genetic modifications. In view of that lignocellulosic hydrolysates contain numerous amounts of xylose and glucose, RT1389-3 may directly utilize hemicellulose hydrolysate as feedstocks to produce EGT, which significantly reduce production costs. Therefore, the direct use of hemicellulose hydrolysate as substrates to provide carbon sources would be considered in future studies.

## Conclusions

In this study, we have developed an effective genetic tool to mediate iterative gene editing in *R. toruloides*, which consists of CRISPR-SpCas9 system and Cre-loxp system. This tool has propped up metabolic engineering contents involved in our work. Meanwhile, we also have established a simple high-throughput screening strategy for rapid evaluation of EGT production of corresponding strains. This method not only guarantees 267.4 mg/L EGT production proposed in this study, but also opens up the possibility of further engineering to enhance the performances of EGT-overproducing strains. Unfortunately, we cannot accurately judge about which gene modifications will definitely promote EGT biosynthesis in *R. toruloides* due to ATMT-mediated random integration. Therefore, to carry out metabolic engineering more rationally, it is necessary to establish a better molecular operation platform for *R. toruloides* from two aspects: screening reliable free plasmids or remodeling more effective HR systems. To sum up, this work has laid the technical and preliminary research foundation for the efficient accumulation of EGT with *R. toruloides*, and is conducive to obtaining high-yield EGT industrial strains with independent intellectual property rights.

## Materials and methods

### Strains and growth media

*Escherichia coli* DH5α, *Agrobacterium tumefaciens* GV3101 and *R. toruloides* used in this study are listed in Table 1. *E. coli* grown at 37 °C in LB medium (1 L) (10 g NaCl, 10 g tryptone, 5 g yeast extract) was used for plasmid construction and storage. When required, the medium was added with 150 μg/mL hygromycin or 50 μg/mL kanamycin. *A. tumefaciens* grown at 28 °C in LB medium with the addition of 50 μg/mL gentamicin was used for plasmid transfer as described previously [46]. *R. toruloides* grown at 30 °C in YPD medium (1 L) (20 g glucose, 10 g peptone, 10 g yeast extract) was prepared for EGT production. When required, the medium was added with 50 μg/mL hygromycin or 50 μg/mL geneticin [47].

For flask fermentation, a single colony of *R. toruloides* was pre-cultured in 5 mL YPD medium for 24 h and then cultured in 20 mL YPD medium for 24 h until OD_600_ of 16. Subsequently, 5 mL culture grown on the YPD medium was inoculated into 50 mL Z1 fermentation medium (1 L) (25 g glucose, 10 g peptone, 10 g yeast extract, 5 g MgSO_4_·7H_2_O, 3 g glutamic acid, 2.5 g KH_2_PO_4_, 0.5 g (NH_4_)_2_SO_4_, 0.278 g FeSO_4_·7H_2_O, 0.1 g thiamine hydrochloride, 0.085 g MnSO_4_·H_2_O, 0.6 mg biotin), Z2 fermentation medium (1 L) (25 g glucose, 15 g peptone, 10 g yeast extract, 5 g MgSO_4_·7H_2_O, 2.5 g KH_2_PO_4_, 0.3 g FeSO_4_·7H_2_O, 0.1 g MnSO_4_·H_2_O, 0.5 mg biotin) with the addition of multi-vitamin solution (200 μg/L pyridoxine, 400 μg/L riboflavin, 400 μg/L niacin, 400 μg/L thiamine, 2000 μg/L inositol, 200 μg/L p-aminobenzoic acid, 400 μg/L calcium pantothenate) and Z3 fermentation medium (1 L) (50 g glucose, 0.75 g yeast extract, 10 g peptone, 1.5 g MgSO_4_·7H_2_O, 0.4 g KH_2_PO_4_, 40 mg CaCl_2_, 5 mg FeSO_4_·7H_2_O, 1 mg ZnSO_4_·7H_2_O, 7.6 mg MnSO_4_·H_2_O, 500 µg biotin, 200 µg pyridoxine, 400 µg riboflavin, 400 µg niacin, 400 µg thiamine, 2000 µg inositol, 200 µg p-aminobenzoic acid, 400 µg calcium pantothenate) at 30 °C on a rotary shaker at 220 rpm.

### Plasmid construction

Plasmids used in this study were listed in Table S2, which were constructed via restriction enzymes and T4 DNA ligase (Thermo Fisher Scientific, America) or One Step Cloning Kit (YEASEN, China) procedures (Additional file 1). The basic plasmids pRtCas9 (previously known as NM9-SpCas9-NLS3) and pRtgRNA-NT (previously known as NM1-5S-tRNA-SgH) were purchased from addgene (https://www.addgene.org/) [35]. The codon optimized *Cre* gene was synthesized by Genscript (Nanjing, China). To develop an iterative genome editing tool, the promoter *Pgal1* (promoter of galactokinase), the *Cre* gene, the terminator *Thsp* (terminator of *hsp*70) were cloned into the plasmid pRtCas9, respectively, generating pRtCas9-gal-Cre. Two identical *loxp* sites (5’-ATAACTTCGTATAGCATACATTATACGAAGTTAT-3’) were inserted into two flanks of gRNA and selective marker gene on the plasmid pRtgRNA-NT, generating pRtgRNA-NT-loxp. For subsequent plasmid-based genome editing, we did just sufficient plasmid design and implementation to obtain correct plasmids based on the plasmid pRtgRNA-NT-loxp. All primers used in this study synthesized by Tsingke (Shanghai, China) were listed in Table S1.

### Agrobacterium tumefaciens-mediated transformation (ATMT)

The standard transformation procedure of *R. toruloides* was performed as described previously [46]. In short, overnight cultures of *R. toruloides* strains and *A. tumefaciens* strains were diluted to OD_600_ of 0.6. Subsequently, 100 μL *R. toruloides* culture and equivoluminal *A. tumefaciens* culture were mixed and coated on induction medium (IM) plate. After 2–3 days of incubation in a 28 °C incubator, the colony on IM plate was collected with 1 mL sterile water, centrifuged and re-suspended in 200 μL sterile water. 100 μL cells was then plated on selective YPD media and incubated for 4–5 days until the transformants appeared. To obtain the correct transformants, transformants grown on selective YPD media were randomly selected for genomic DNA extraction, which served as a template for PCR amplification using 2xEs Taq MasterMix (CWBIO, China) or KOD One (TOYOBO, Japan), and the PCR products were verified by agarose gel electrophoresis and sanger sequencing (Tsingke, China).

### Resistance curing

The positive mutants, which contained pRtgRNA-NT-loxp-derived plasmids, were inoculated into 5 mL induction medium (20 g/L peptone, 20 g/L galactose, 10 g/L yeast extract, 10 g/L raffinose) for 24 h, and then diluted and coated on induced plate containing G418 resistance for 3 days. Finally, a number of above colonies were verified by PCR identification using the primer pairs Hyg-F/Hyg-R, and the non-resistant colonies were preserved for further research.

### Design of CRISPR/SpCas9 target

The 20 nt spacer sequences in this study were designed by the CHOPCHOP online website (http://chopchop.cbu.uib.no/) (Table S3). Taking *Ku70* gene as an example, first, the relevant parameters were set (Species: *Saccharomyces cerevisiae* sacCer3/A288c; tool: CRISPR/Cas9; purpose: knock out; 5'-PAM: NGG; length: 20). Second, *Ku70* gene was pasted as FASTA format. Third, the program was operated to obtain arranged spacers.

### High-performance liquid chromatography (HPLC) analysis to measure EGT

To accurately quantify EGT, the fermentation culture, harvested on the 7th day, was processed according to two methods of intracellular and extracellular treatment. The intracellular treatment method was performed as described previously [48]. As for extracellular treatment method, a total of 2 mL fermentation culture was centrifuged (12,000 × g, 5 min), and 1 mL liquid supernatant was then transferred to 1.5 mL Eppendorf tube, which was centrifuged (12000 × g, 30 min). 100 μL final supernatant was filtrated using 0.22 μm filter membrane. The sample was detected using HPLC system (Agilent 1260) equipped with a Diamonsil Plus 5 μm C18-A column (250 × 4.6 mm) and the detection wavelength was 254 nm. The mobile phase was acetonitrile–water (3:97 v/v) with a flow rate of 0.6 mL/min at room temperature [48]. The standard of EGT was dissolved in 70% acetonitrile to prepare the standard curves. Product contents were expressed as mg per liter (mg/L).

### High-throughput screening method of EGT

The DNA sequence of *Burkholderia* sp. HME13 ergothionase (GenBank: AB699692.1) was synthesized and inserted into the plasmid pet28a by Genscript (Nanjing, China), generating the plasmid pet28a-ergothionase. The BL21 transformant harboring the pet28a-ergothionase was grown in 50 mL LB medium at 37 °C until OD_600_ = 0.8, and then induced with 0.1 mM IPTG at 18 °C for 16–20 h. Subsequently, the cells were washed and re-suspended in 15 mL PBS buffer, and then treated with ultrasonication at 300 V (5 s sonication; 5 s interval; 99 times). Finally, the solution was stored at −40 °C with a final concentration of 20% glycerol. The detailed high-throughput screening process was as follows: A series of *R. toruloides* transformants were inoculated into 96-well plates with 600 μL modified Z2 medium at 30 °C for 7 days. Then, 500 μL of the above cell cultures was collected by centrifugation to obtain the EGT supernatants (12,000 × g, 30 min). Subsequently, 10 μL of crude ergothionase solution was added to the above supernatants (190 μL) to read the starting absorbance in a microplate reader at 311 nm, and then the reaction solution started an enzymatic reaction at room temperature for 30 min to read the ending absorbance. The absorbance change was defined as the difference value between the starting and ending absorbance.

### Supplementary Information

Additional file 1.

## Data Availability

All data generated or analyzed during this study are included in this published article and its additional information files.

## Abbreviations

EGT: Ergothioneine
RT1389: *Rhodotorula toruloides* 2.1389
CACR: CRISPR-assisted Cre recombination
ROS: Reactive oxygen species
SAM: S-adenosylmethionine
His: Histidine
Cys: Cysteine
Glu: Glutamic acid
HCO: Hercynylcysteine sulfoxide
PLP: Pyridoxal phosphate
Met: Methionine
TRA: β-(1,2,4,-Triazol-3-yl)-DL-alanine
MVA: Mevalonic acid
ATMT: *Agrobacterium* *tumefaciens*-Mediated transformation
T-DNA: Transfer DNA
NHEJ: Non-homologous end joining
p2A1: *Porcine teschovirus*-1 2A
p2A2: *Zaire ebolavirus* 2A
HR: Homologous recombination
HPLC: High-performance liquid chromatography

## References

[1] Apparoo Y, Phan CW, Kuppusamy UR, Sabaratnam V (2022). Ergothioneine and its prospects as an anti-ageing compound. Exp Gerontol..

[2] Cheah IK, Halliwell B (2012). Ergothioneine; antioxidant potential, physiological function and role in disease. Biochim Biophys Acta-Mol Basis Dis.

[3] Han Y, Tang X, Zhang Y, Hu X, Ren LJ (2021). The current status of biotechnological production and the application of a novel antioxidant ergothioneine. Crit Rev Biotechnol.

[4] Qiu Y, Chen Z, Su E, Wang L, Sun L, Lei P, Xu H, Li S (2021). Recent strategies for the biosynthesis of ergothioneine. J Agric Food Chem.

[5] Halliwell B, Cheah IK, Tang RMY (2018). Ergothioneine–a diet-derived antioxidant with therapeutic potential. FEBS Lett.

[6] Gründemann D, Harlfinger S, Golz S, Geerts A, Lazar A, Berkels R, Jung N, Rubbert A, Schömig E (2005). Discovery of the ergothioneine transporter. Proc Natl Acad Sci.

[7] Gründemann D (2012). The ergothioneine transporter controls and indicates ergothioneine activity—a review. Prev Med.

[8] Yang NC, Lin HC, Wu JH, Ou HC, Chai YC, Tseng CY, Liao JW, Song TY (2012). Ergothioneine protects against neuronal injury induced by β-amyloid in mice. Food Chem Toxicol.

[9] Einar S, Filip O, Sophie H, Ulrika E, Marju OM, Céline F, Olle M (2020). Ergothioneine is associated with reduced mortality and decreased risk of cardiovascular disease. Heart.

[10] Samuel P, Tsapekos M, de Pedro N, Liu AG, Casey Lippmeier J, Chen S (2022). Ergothioneine mitigates telomere shortening under oxidative stress conditions. J Diet Suppl.

[11] Yen MT, Chang YH, Huang SJ, Cheng MC, Mau JL (2018). Extraction of ergothioneine from pleurotus eryngii and P-citrinopileatus (*Agaricomycetes*) and preparation of its product. Int J Med Mushrooms.

[12] Xu J, Yadan JC (1995). A new and convenient synthesis of imidazole-2-thiones from imidazoles. Synlett.

[13] Beliaeva MA, Burn R, Lim D, Seebeck FP (2021). In vitro production of ergothioneine isotopologues. Angew Chem-Int Edit.

[14] Melville DB, Genghof DS, Inamine E, Kovalenko V (1956). Ergothioneine in microorganisms. J Biol Chem.

[15] Genghof DS, Vandamme O (1964). Biosynthesis of ergothioneine and hercynine by mycobacteria. J Bacteriol.

[16] Genghof DS (1970). Biosynthesis of ergothioneine and hercynine by fungi and actinomycetales. J Bacteriol.

[17] Vit A, Misson L, Blankenfeldt W, Seebeck FP (2014). Crystallization and preliminary X-ray analysis of the ergothioneine-biosynthetic methyltransferase EgtD. Acta Crystallogr F.

[18] Jones GW, Doyle S, Fitzpatrick DA (2014). The evolutionary history of the genes involved in the biosynthesis of the antioxidant ergothioneine. Gene.

[19] Goncharenko KV, Vit A, Blankenfeldt W, Seebeck FP (2015). Structure of the sulfoxide synthase EgtB from the ergothioneine biosynthetic pathway. Angew Chem-Int Edit.

[20] Vit A, Mashabela GT, Blankenfeldt W, Seebeck FP (2015). Structure of the ergothioneine-biosynthesis amidohydrolase EgtC. ChemBioChem.

[21] Khonde PL, Jardine A (2015). Improved synthesis of the super antioxidant, ergothioneine, and its biosynthetic pathway intermediates. Org Biomol Chem.

[22] Pluskal T, Ueno M, Yanagida M (2014). Genetic and metabolomic dissection of the ergothioneine and selenoneine biosynthetic pathway in the fission yeast, S. pombe, and construction of an overproduction system. PLoS ONE.

[23] Osawa R, Kamide T, Satoh Y, Kawano Y, Ohtsu I, Dairi T (2018). Heterologous and high production of ergothioneine in *Escherichia coli*. J Agric Food Chem.

[24] Chen ZH, He YZ, Wu XY, Wang L, Dong ZY, Chen XZ (2022). Toward more efficient ergothioneine production using the fungal ergothioneine biosynthetic pathway. Microb Cell Fact.

[25] Zhang L, Tang J, Feng M, Chen S (2023). Engineering methyltransferase and sulfoxide synthase for high-yield production of ergothioneine. J Agric Food Chem.

[26] van der Hoek SA, Darbani B, Zugaj KE, Prabhala BK, Biron MB, Randelovic M, Medina JB, Kell DB, Borodina I (2019). Engineering the yeast *Saccharomyces cerevisiae* for the production of L-(+)-Ergothioneine. Front Bioeng Biotechnol.

[27] van der Hoek SA, Rusnák M, Wang G, Stanchev LD, de Fátima AL, Jessop-Fabre MM, Paramasivan K, Jacobsen IH, Sonnenschein N, Martínez JL, Darbani B, Kell DB, Borodina I (2022). Engineering precursor supply for the high-level production of ergothioneine in *Saccharomyces cerevisiae*. Metab Eng.

[28] Wen Z, Zhang S, Odoh CK, Jin M, Zhao ZK (2020). *Rhodosporidium toruloides*
-a potential red yeast chassis for lipids and beyond. FEMS Yeast Res.

[29] Yu Y, Shi S (2023). Development and perspective of *Rhodotorula toruloides* as an efficient cell factory. J Agric Food Chem.

[30] Park YK, Nicaud JM, Ledesma AR (2018). The engineering potential of *Rhodosporidium toruloides* as a workhorse for biotechnological applications. Trends Biotechnol.

[31] Zhu Z, Zhang S, Liu H, Shen H, Lin X, Yang F, Zhou YJ, Jin G, Ye M, Zou H, Zhao ZK (2012). A multi-omic map of the lipid-producing yeast *Rhodosporidium toruloides*. Nat Commun.

[32] Guo X, Bai Z, Zhang Y, Zhao H, Shi S (2023). Mining and application of constitutive promoters from *Rhodosporidium toruloides*. AMB Express.

[33] Johns AMB, Love J, Aves SJ (2016). Four inducible promoters for controlled gene expression in the oleaginous yeast *Rhodotorula toruloides*. Front Microbiol.

[34] Sun W, Yang X, Wang X, Jiao X, Zhang S, Luan Y, Zhao ZK (2018). Developing a flippase-mediated maker recycling protocol for the oleaginous yeast *Rhodosporidium toruloides*. Biotechnol Lett.

[35] Schultz JC, Cao M, Zhao H (2019). Development of a CRISPR/Cas9 system for high efficiency multiplexed gene deletion in *Rhodosporidium toruloides*. Biotechnol Bioeng.

[36] Jiao X, Zhang Y, Liu X, Zhang Q, Zhang S, Zhao ZK (2019). Developing a CRISPR/Cas9 system for genome editing in the basidiomycetous yeast *Rhodosporidium toruloides*. Biotechnol J.

[37] Otoupal PB, Ito M, Arkin AP, Magnuson JK, Gladden JM, Skerker JM (2019). Multiplexed CRISPR-Cas9-based genome editing of *Rhodosporidium toruloides*. Msphere.

[38] Liu Y, Koh CMJ, Sun L, Hlaing MM, Du M, Peng N, Ji L (2013). Characterization of glyceraldehyde-3-phosphate dehydrogenase gene RtGPD1 and development of genetic transformation method by dominant selection in oleaginous yeast Rhodosporidium toruloides. Appl Microbiol Biotechnol.

[39] Liu H, Jiao X, Wang Y, Yang X, Sun W, Wang J, Zhang S, Zhao ZK (2017). Fast and efficient genetic transformation of oleaginous yeast *Rhodosporidium toruloides* by using electroporation. FEMS Yeast Res.

[40] Jiao X, Zhang Q, Zhang S, Yang X, Wang Q, Zhao ZK (2018). Efficient co-expression of multiple enzymes from a single promoter mediated by virus 2A sequence in the oleaginous yeast
*Rhodosporidium toruloides*. FEMS Yeast Res.

[41] Chen R, Gao J, Yu W, Chen X, Zhai X, Chen Y, Zhang L, Zhou YJ (2022). Engineering cofactor supply and recycling to drive phenolic acid biosynthesis in yeast. Nat Chem Biol.

[42] Muramatsu H, Matsuo H, Okada N, Ueda M, Yamamoto H, Kato S-i, Nagata S (2013). Characterization of ergothionase from Burkholderia sp. HME13 and its application to enzymatic quantification of ergothioneine. Appl Microbiol Biotechnol.

[43] Xiong KX, Dong NH, Yang B, Chen YX, Liang HP, Lin XP, Zhang SF (2023). Ergothioneine yield of
*Rhodotorula* species positively correlated with hydrogen peroxide tolerance. Food Biosci.

[44] Xiong KX, Guo H, Xue SY, Dai YW, Dong L, Ji CF, Zhang SF (2023). Cost-effective production of ergothioneine using
*Rhodotorula mucilaginosa* DL-X01 from molasses and fish bone meal enzymatic hydrolysate. Bioresource Technol.

[45] Tiukova IA, Brandenburg J, Blomqvist J, Sampels S, Mikkelsen N, Skaugen M, Arntzen MO, Nielsen J, Sandgren M, Kerkhoven EJ (2019). Proteome analysis of xylose metabolism in *Rhodotorula toruloides* during lipid production. Biotechnol Biofuels.

[46] Wang Y, Zhang S, Pötter M, Sun W, Li L, Yang X, Jiao X, Zhao ZK (2016). Overexpression of Δ12-fatty acid desaturase in the oleaginous yeast *Rhodosporidium toruloides* for production of linoleic acid-rich lipids. Appl Biochem Biotechnol.

[47] Lin X, Wang Y, Zhang S, Zhu Z, Zhou YJ, Yang F, Sun W, Wang X, Zhao ZK (2014). Functional integration of multiple genes into the genome of the oleaginous yeast *Rhodosporidium toruloides*. FEMS Yeast Res.

[48] Xiong LB, Xie ZY, Ke J, Wang L, Gao B, Tao XY, Zhao M, Shen YL, Wei DZ, Wang FQ (2022). Engineering *Mycolicibacterium neoaurum* for the production of antioxidant ergothioneine. Food Bioeng.

