# Supplementary material for: Engineering non-conventional yeast Rhodotorula toruloides for ergothioneine production
Source: Biotechnol Biofuels Bioprod. 2024 May 13;17:65. doi: 10.1186/s13068-024-02516-2 (PMC11089744; doi:10.1186/s13068-024-02516-2)
Supplement: Supplementary file 1 — Additional file 1. [file 13068_2024_2516_MOESM1_ESM.docx]

**Engineering non-conventional yeast *Rhodotorula toruloides* for ergothioneine production**

Ke Liu^1^, Gedan Xiang^1^, Lekai Li^1^, Tao Liu^1^, Jie Ke^1^, Liangbin Xiong^1,2^, Dongzhi Wei^1^ and Fengqing Wang^1*^

**Figure S1** HPLC results of the EGT product of RT1389 in original Z1 fermentation medium. The green arrow points to the peak time of EGT.

**Figure S2** Growth curve (**A**) and EGT production (**B**) of RT1389 in original Z2 fermentation medium.

**Figure S3** Effect of supplementation of low concentration gradient of Cys on EGT production of RT1389 strain in Z2 medium with 2 g/L Met supplementation.

**Figure S4** PCR screening and DNA sequencing of the desired gene editing events in RT1389-1. **A** Result of *SPE2* gene editing events. **B** Result of *PAL* gene editing events. *Mar* DNA ladder marker. *Con* Control group. *S* Experimental sample with single sgRNA expression cassette. *T* Experimental sample with two sgRNA expression cassette.

**Figure S5** Growth (**A**) and EGT production (**B**) of RT1389 and RT1389-1 in original Z2 fermentation medium.

**Figure S6** qPCR assay of *RtEGT1* and *RtEGT2* genes in strains E1-E2-1 and E1-E2-5 at 120 h of fermentation. **A** original transcriptional data of *RtEGT1* and *RtEGT2* provided by Personalbio (Shanghai, China). The relative gene expression was normalized to the abundance of 26S gene (RHTO_01599). **B** The transcriptional expression level of genes *RtEGT1* and *RtEGT2* of strains E1-E2-1 and E1-E2-5. *RtEGT1* and *RtEGT2* genes mRNA expression level of the RT1389-1 strain was regarded as 1.

**Figure S7** Flowchart for high-throughput analysis of EGT production in *R. toruloides* engineering strain.

**Figure S8** Results of the absorbance change of 192 *R. toruloides* engineering strain after being catalyzed by ergothionase crude extract at room temperature for 30 min. The highlighted values correspond to the selected *R. toruloides* engineering strains for subsequent fermentation testing in this study.

**Figure S9** Growth curve (**A**) and EGT production (**B**) of RT1389-3 in modified Z2 medium containing xylose.

**Table S1** Primers and related DNA sequences used in this study.

**Table S2** Plasmids used in this study.

**Table S3** Results of single and double target gene editing mediated by CACR system of RT1389-1 in this study.

**Table S4** Results of sequence alignment analyses with the homologs from *S. cerevisiae* S288C.

**Fig. S1**

**

**

**Fig. S2**

**
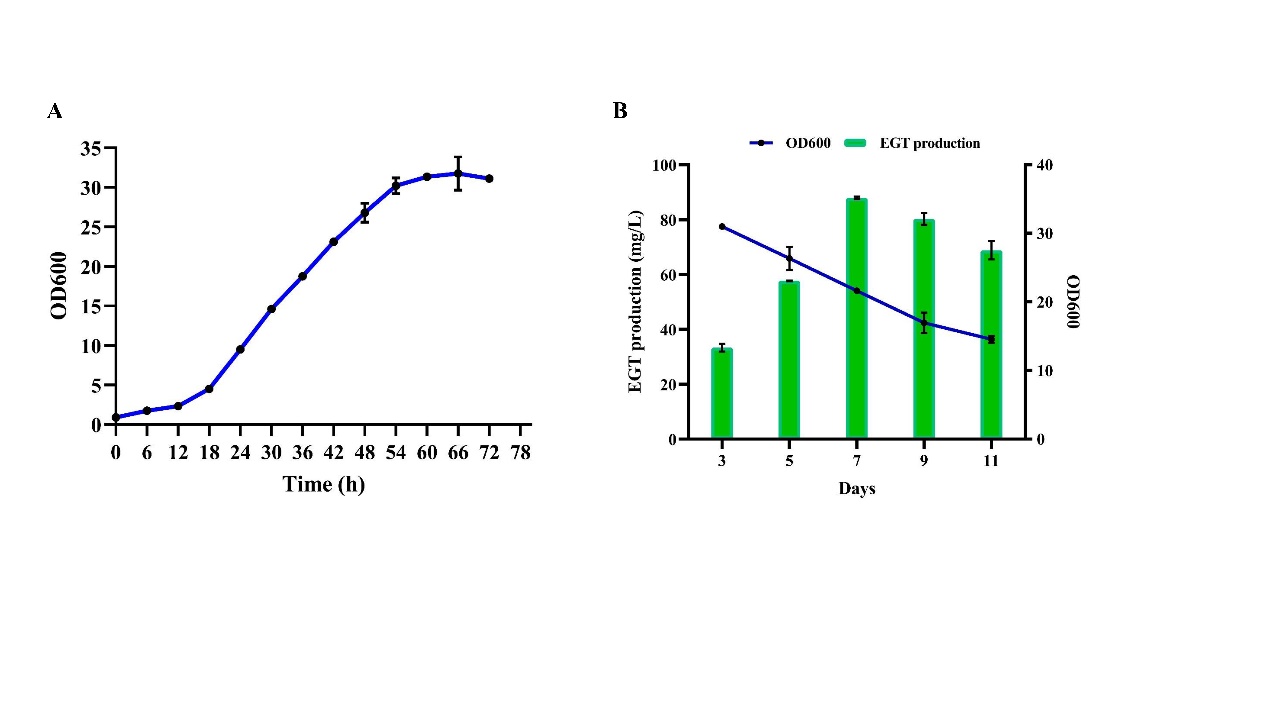
**

**Fig. S3**

**
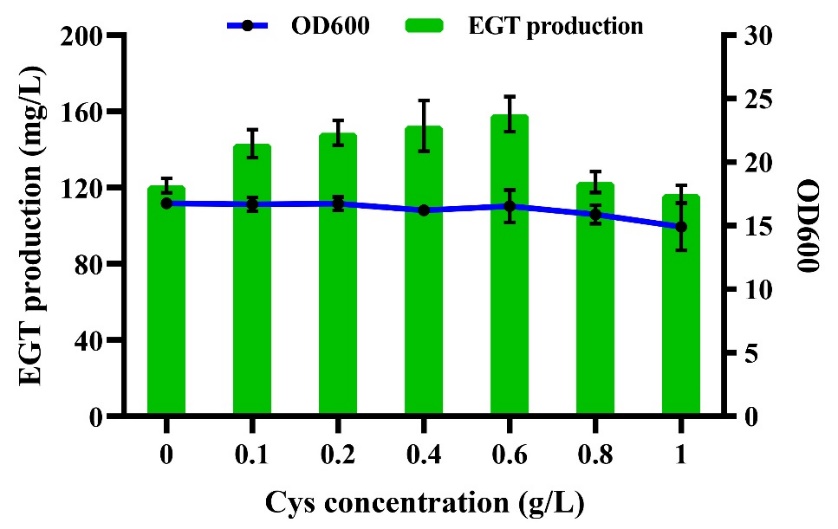
**

**Fig. S4**

**
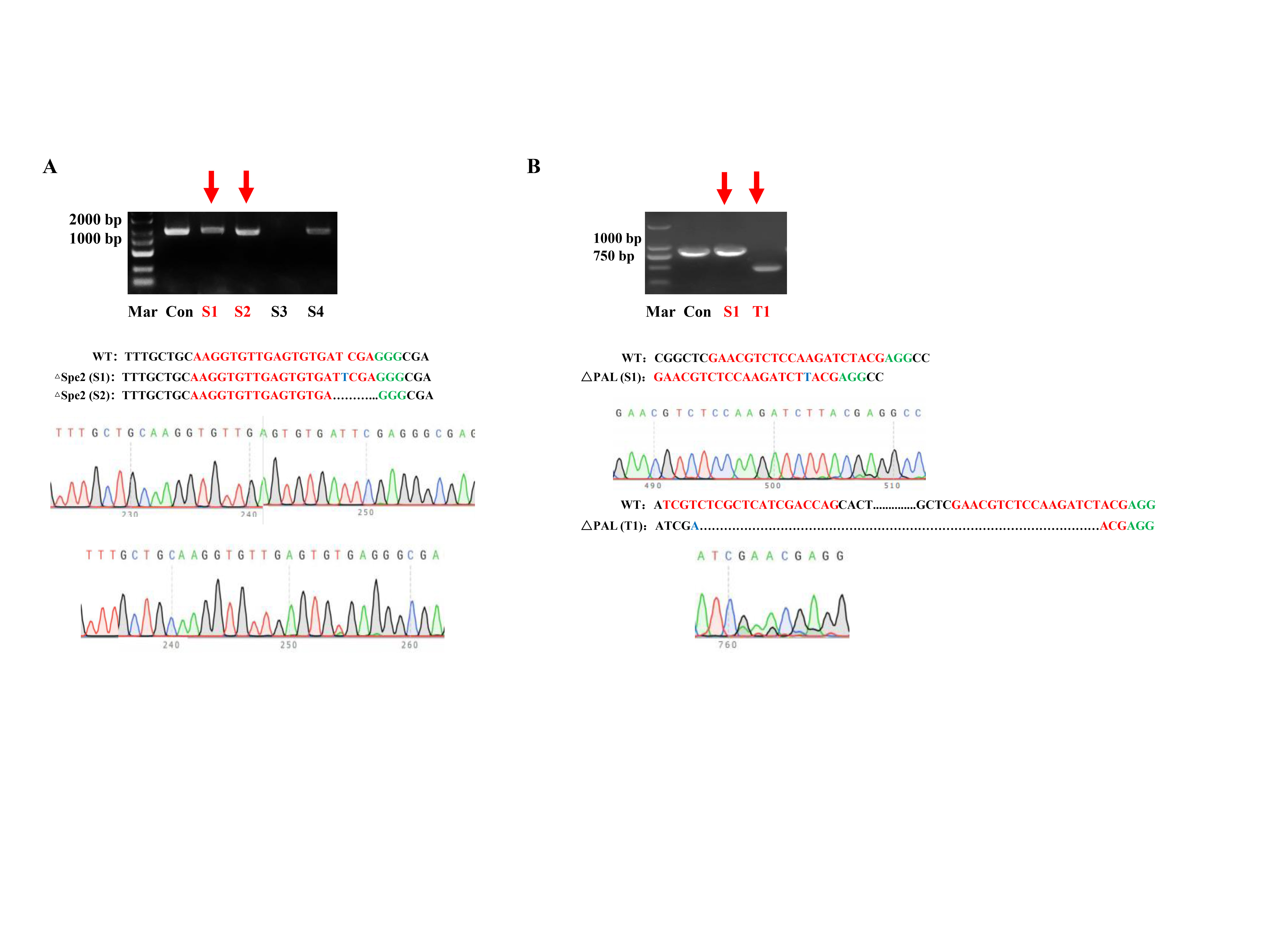
**

**Fig. S5**

**
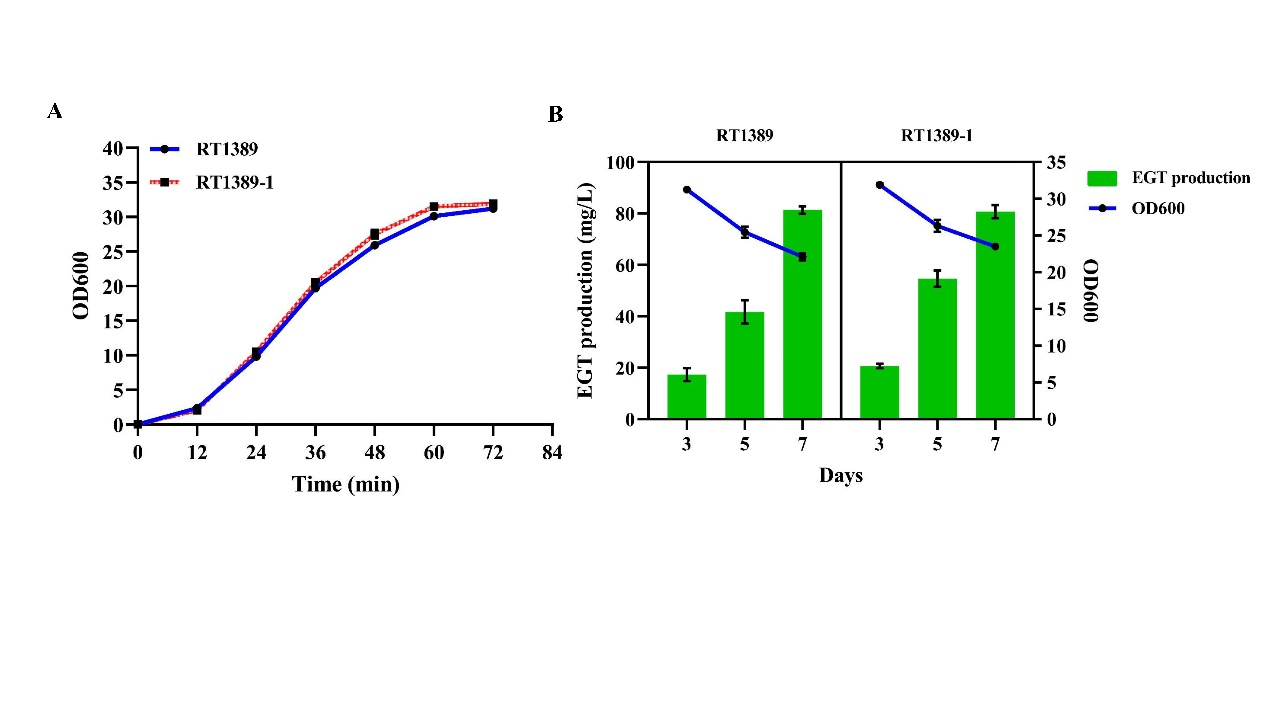
**

**Fig. S6**

**
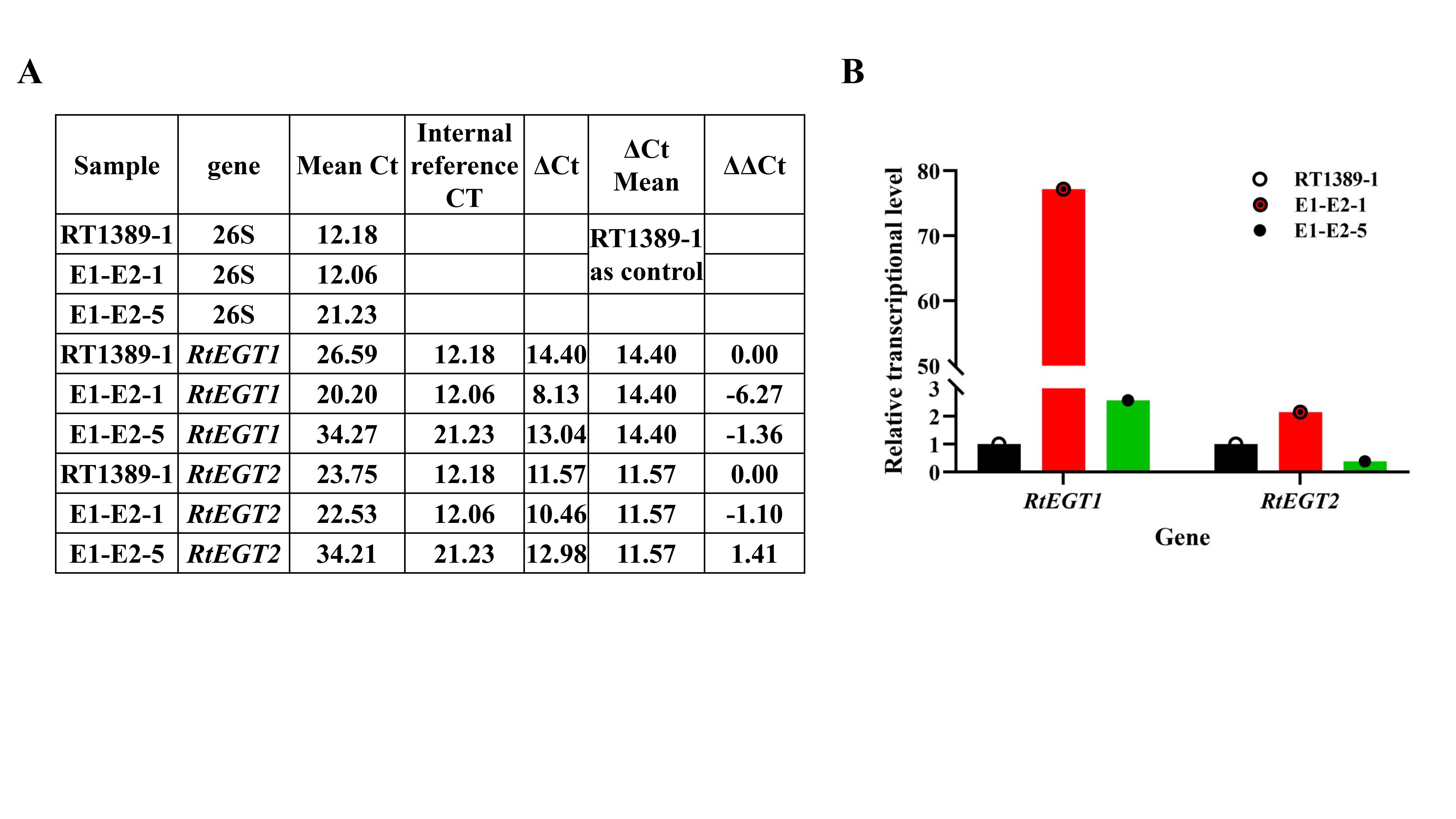
**

**Fig. S7**

**

**

**Fig. S8**

**

**

**Fig. S9**

**
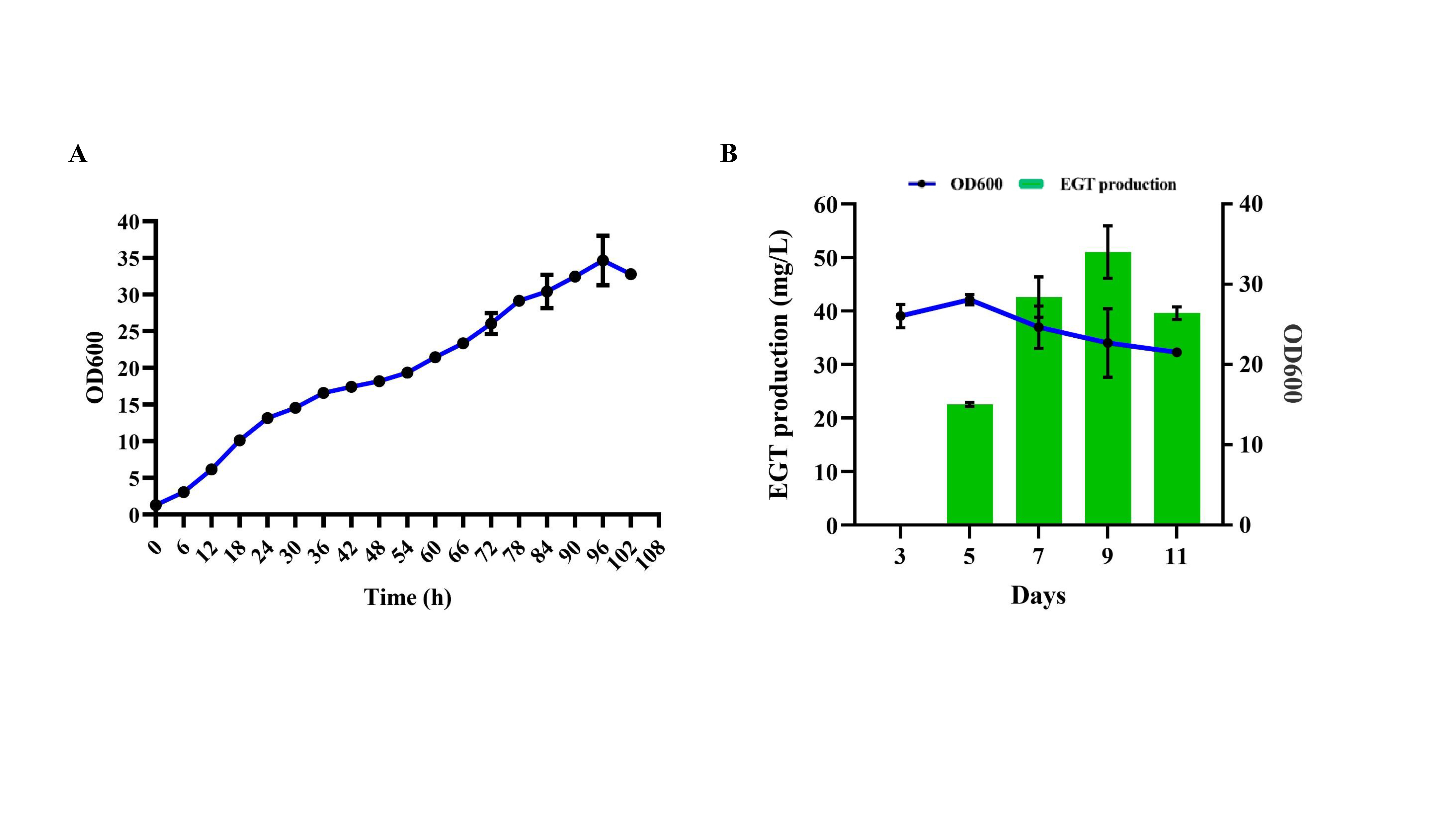
**

**Table S1**

| Name | Sequence (5’-3’) |
| --- | --- |
| Ku70-F | CTACAAAGACAGCGTGCGTCGG |
| Ku70-R | CCTTGAGATTCGCGACCGTGAGTT |
| Hyg-F | ATCGGAGAGTTCTCGGAATC |
| Hyg-R | GCCAAATGTTTGAACGATCC |
| crKu70-1-F | AGGTTAGCGAGACAAGACTGGTTTTAGAGCTAGAAATAGCAAGTT |
| crKu70-1-R | CAGTCTTGTCTCGCTAACCTCGACGGTGAAGGGGATCGAA |
| crKu70-2-F | GCTGTACGAGACGATCCATGGTTTTAGAGCTAGAAATAGCAAGTT |
| crKu70-2-R | CATGGATCGTCTCGTACAGCCGACGGTGAAGGGGATCGAA |
| 2crgene-F | GTCGGTGCTTTTTTTTTGGTATCTGCGGCCATACCGCGAT |
| 2crgene-R | CGTAGTTCTGCAGCCCGGGTACCAAAAAAAAAGCACCGACTCGGTGCCAC |
| crSpe2-1-F | AAGGTGTTGAGTGTGATCGAGTTTTAGAGCTAGAAATAGCAAGTT |
| crSpe2-1-R | TCGATCACACTCAACACCTTCGACGGTGAAGGGGATCGAA |
| crSpe2-2-F | CTGCGTTCCCGACTCCCCGGGTTTTAGAGCTAGAAATAGCAAGTT |
| crSpe2-2-R | CCGGGGAGTCGGGAACGCAGCGACGGTGAAGGGGATCGAA |
| crPAL-1-F | GAACGTCTCCAAGATCTACGGTTTTAGAGCTAGAAATAGCAAGTT |
| crPAL-1-R | CGTAGATCTTGGAGACGTTCCGACGGTGAAGGGGATCGAA |
| crPAL-2-F | CTGGTCGATGAGCGAGACGAGTTTTAGAGCTAGAAATAGCAAGTT |
| crPAL-2-R | TCGTCTCGCTCATCGACCAGCGACGGTGAAGGGGATCGAA |
| Spe-F | GGTCCTTTCGAAGGTTCGTCGTCCG |
| Spe-R | TGAGGCCCAAGTCGTCCGAGAGTTT |
| PAL-F | AAGACCCCTCGCTCTCCTACCACT |
| PAL-R | CGAGCATCTTGAGGAGGACGTTGTT |
| Ppgk-F | GTAAAACGACGGCCAGTGAACCAGACGGACCTTGAGAACCCTC |
| Ppgk-R | GAGGTACCGGGCTCGAATTCGGTGGGCTGCACGGGGGA |
| Egt1-F | CACCGAATTCATGCCCACCGCACTCACAGA |
| Egt1-R | TGCCAAATGTTTGAACGATCTAAGTATCAGACGTCGTAGGCGATC |
| Egt1-GF | TCGCCTACGACGTCTGAGAGCCCGGTACCTCGCGAATG |
| Egt1-GR | CGGTGGGCATGAATTCGGTGGGCTGCACG |
| NOS-F | ACCTCGCGAATGCATCTGATCGTTCAAACATTTGGCAATAAA |
| NOS-R | CCTGTTAAGTATCTAGAGATCTAGTAACATAGATGACACCGC |
| PGK-xbaI-F | ATTATACGAAGTTATTCCTCTCCAGACGGACCTTGAGAACCCTC |
| PGK-xbaI-R | CATGCCTGCAGGTCGACTCTAGAGATCTAGTAACATAGATGACACCGCGCG |
| Met14-F | CCGTGCAGCCCACCGAATTCATGTCCGCCCCTCACAGCAC |
| Met14-R | GTTTGAACGATCTAAGTATCACTTGTGGCATCCTCGAGGC |
| SAM2-F | CACCGAATTCATGTCCAACACTTTCCTCTTCACCT |
| SAM2-R | GATCTAAGTATTAGAGCTGGAGCTTCTTGGGAACC |
| SAH1-F | CACCGAATTCATGGTCTACAAGGTGTACGTGCAGG |
| SAH1-R | GATCTAAGTATTAGTAGCGGTAGTGCTCGGGCTTG |
| ADO1-F | CACCGAATTCATGTCCAACTCGCCCCTCCT |
| ADO1-R | GATCTAAGTATTAGTCCGGTTGAACGATCCAGCG |
| Met14-GF | GAGGATGCCACAAGTGATACTTAGATCGTTCAAACATTTGGCAATAAAG |
| Met14-GF | GTGCTGTGAGGGGCGGACATGAATTCGGTGGGCTGCACGG |
| SAM2-GF | CCAGCTCTAATACTTAGATCGTTCAAACATTTGGC |
| SAM2-GR | TGTTGGACATGAATTCGGTGGGCTGCACG |
| SAH1-GF | CCGCTACTAATACTTAGATCGTTCAAACATTTGGC |
| SAH1-GR | TGTAGACCATGAATTCGGTGGGCTGCACG |
| ADO1-GF | ACCGGACTAATACTTAGATCGTTCAAACATTTGGC |
| ADO1-GR | AGTTGGACATGAATTCGGTGGGCTGCACGG |
| Pgpd-F | TTAAACTAAGTATCGGATGGAGTTCGACGTTCTCCTC |
| Pgpd-R | AGTCGACGGGCCCGGGATCCTGTGACTGATCTGGTGTTGTTC |
| Egt2-F | ACACCAGATCAGTCACAGGAATGACGAAGACACACTCGTTGTTCT |
| Egt2-R | CAGTCGACGGGCCCGGGATCCCTACGGCTGCTCCGCGTCAG |
| hsp-F | AGAGGCCTGCATGCAAGACGATTCCGCCCCGTCTCAC |
| hsp-R | ACCATGATTACGCCAAGCTTCGCGCACTTCTCTGCACTGC |
| GPD-xbaI-F | ATCTATGTTACTAGATCTCTTGGAGTTCGACGTTCTCCTCGCTCC |
| GPD-xbaI-R | CATGCCTGCAGGTCGACTCTAGACGCGCACTTCTCTGCACTGCAT |
| 1301-E1-F | ACTCTTGACCATGGTAGATCTATGCCCACCGCACTCACAGA |
| 1301-E1-R | TCGGGGAAATTCGAGCTGGTCACCTCAGACGTCGTAGGCGATCCT |
| 1301-E2-F | GGACTCTTGACCATGGTAGATCTATGACGAAGACACACTCGTTGTTCTTTTAC |
| 1301-E2-R | GGGGAAATTCGAGCTGGTCACCCTACGGCTGCTCCGCGTCAG |
| NEGT1-F | CATTATACGAAGTTATTCCTCTTGAGACTTTTCAACAAAGGGTAATATCCGG |
| NEGT2-R | TTGCATGCCTGCAGGTCGACTCTAGAGATCTAGTAACATAGATGACACCGCGC |
| NEGT2-F | ATCTATGTTACTAGATCTCTTGAGACTTTTCAACAAAGGGTAATATCCGG |
| P2A1-F1 | TGAAGCAGGCTGGAGACGTGGAGGAGAACCCTGGACCTATGACGAAGACACACTCGTTGTTCTTTTAC |
| P2A1-R | GGGGAAATTCGAGCTGGTCACCCTACGGCTGCTCCGCGTCAG |
| P2A1-F2 | GGATCGCCTACGACGTCGGAAGCGGAGCTACTAACTTCAGCCTGCGGATCGCCTACGACGTCGGCTCGGGCGCTACCAACTTCTCGCTCC |
| P2A2-F1 | TCAAGCAGGCTGGCGACGTCGAAGAGAACCCTGGCCCTATGACGAAGACACACTCGTTGTTCTTTTAC |
| P2A2-F2 | GGATCGCCTACGACGTCGGCTCGGGCGCTACCAACTTCTCGCTCCTCAAGCAGGC |
| MET14-SAM2-F1 | CTCGAGGATGCCACAAGGGAAGCGGAGCTACTAACTTCAGCCTGCTGAAGCAGGCTGGAGACGTGGAGGAGAACCCTGGACCTATGTCCAACACTTTCCTCTTCACCT |
| MET14-SAM2-GF | AAGAAGCTCCAGCTCTAATACTTAGATCGTTCAAACATTTGGCAATAAAG |
| MET14-SAM2-GR | TTAGTAGCTCCGCTTCCCTTGTGGCATCCTCGAGGCG |
| 14-2-SAH1-F1 | GGAAGCGGAGCTACTAACTTCAGCCTGCTGAAGCAGGCTGGAGACGTGGAGGAGAACCCTGGACCTATGGTCTACAAGGTGTACGTGCAGG` |
| 14-2-SAH1-GR | TTAGTAGCTCCGCTTCCGAGCTGGAGCTTCTTGGGAA |
| Pgal1 | TTGGCGAGGATGGCGGACGAGGCGAATGGGTCGGAAGGCATCGTCGTCTCCATCTCGACTCGACACGGTCTCGCCTGTTCCTCCTTCAACTCGCGCTCCCAATCTCCATCTACTGCGTCCGCCACGAATTCGTTCCCGCCCAGGAGTGCGGTGATCTCGAAGCCGTCGTGTGAGGCATTCCGAGTCGCCGTCTGTCGCCGCTCGTCCTTCCAGCTCCTTTCGAGCCCAGCGAGCCCGTTCACTCCTCCCGTGAATGCCGCAAAGTCATTCTCTGCGCGCGCGGCGGAGGGCGAAGCGTCGGGTCGGAAGCTGGTGGAGCCGGAGGGGAACGACGAGGACGAGGACGAGGGGGCGAGGTGGTCGAGGTGGGCGTGGTCGCTGGCATGAGCAAAGCGCTGGACAGCAGCGGGCAGCCCCTTCGAGTCCATTCCAGCGACAGCGCGGCGAGGCAGAGCGGACGAGAGGGAAGGACTGGTTGGTATTCAGTCACAATACAAGCAATACACTCCAGTTTGTATTGGTTGCTGATGACAACTCGCATTACAACCGCGTTTGTATTGCAAAGGAATACAAAGTTGATGACGCCGTGCCGTCCCGCTCCCGCTCGCCCATCTTCTTTTTCAATACGCTTGATACGCTTGCTGTGCTCTGCTTGCGACC |
| Cre recombinase | ATGTCCAATCTCCTCACCGTCCACCAGAATCTCCCGGCCCTCCCGGTGGATGCCACGTCCGACGAAGTCCGGAAGAACCTCATGGACATGTTCCGGGACCGCCAGGCCTTTTCGGAGCACACCTGGAAGATGCTCCTCTCGGTCTGTCGCTCGTGGGCGGCCTGGTGTAAGCTGAACAACCGCAAATGGTTCCCGGCCGAGCCCGAAGATGTCCGCGACTACCTCCTGTATCTCCAGGCGCGGGGACTGGCCGTCAAAACGATTCAACAGCACCTCGGCCAGCTCAATATGCTCCATCGGCGCTCCGGACTCCCGCGCCCCTCCGATTCCAATGCGGTCTCCCTCGTCATGCGCCGCATCCGGAAAGAGAACGTCGATGCGGGAGAACGCGCCAAACAAGCGCTCGCGTTCGAGCGCACGGACTTCGACCAGGTGCGCTCCCTCATGGAGAACTCGGACCGGTGCCAAGACATCCGCAACCTGGCGTTCCTCGGCATCGCCTACAACACCCTGCTCCGGATTGCGGAGATCGCCCGCATCCGCGTCAAGGACATCTCGCGCACCGATGGAGGCCGCATGCTGATCCACATCGGCCGCACGAAAACGCTCGTAAGCACGGCGGGAGTCGAAAAGGCGCTCTCCCTCGGCGTCACGAAGCTCGTCGAACGGTGGATCTCGGTCTCGGGCGTGGCGGATGATCCGAACAACTACCTCTTCTGCCGCGTGCGGAAAAATGGAGTGGCGGCCCCCTCGGCCACGTCGCAACTCTCGACAAGGGCGCTGGAAGGCATCTTCGAGGCGACGCACCGCCTCATTTACGGCGCGAAGGACGACTCCGGCCAACGCTATCTCGCGTGGTCCGGACATTCCGCAAGGGTCGGCGCGGCCCGCGATATGGCGAGGGCGGGAGTCTCGATTCCGGAGATCATGCAAGCGGGCGGATGGACCAACGTCAACATCGTCATGAACTACATCCGCACGCTCGACTCGGAAACCGGAGCGATGGTCCGCCTCCTGGAAGACGGCGACTAG |
|  |  |
| loxp | ATAACTTCGTATAGCATACATTATACGAAGTTAT |
| p2A2 | GGAAGCGGAGCTACTAACTTCAGCCTGCTGAAGCAGGCTGGAGACGTGGAGGAGAACCCTGGACCT |
| p2A1 | GGCTCGGGCGCTACCAACTTCTCGCTCCTCAAGCAGGCTGGCGACGTCGAAGAGAACCCTGGCCCT |

**Table S2**

| Plasmids and strains | Characteristics | References or sources |
| --- | --- | --- |
| Plasmids |  |  |
| pRtCas9 | NM9-SpCas9-NLS3 | addgene: 128177 |
| pRtgRNA-NT | NM1-5S-tRNA-SgH | addgene: 128178 |
| pRtCas9-gal-Cre | pRtCas9, Cre | This study |
| pRtgRNA-NT-loxp | pRtgRNA-NT, loxp | This study |
| pRtgRNA-Ku70-1-loxp | pRtgRNA-NT-loxp, CrKu70-1 | This study |
| pRtgRNA-Ku70-2-loxp | pRtgRNA-NT-loxp, CrKu70-2 | This study |
| pRtgRNA-2Ku70-loxp | pRtgRNA-Ku70-1-loxp, CrKu70-2 | This study |
| pRtgRNA-Spe2-1-loxp | pRtgRNA-NT-loxp, CrSpe2-1 | This study |
| pRtgRNA-Spe2-2-loxp | pRtgRNA-NT-loxp, CrSpe2-2 | This study |
| pRtgRNA-PAL-1-loxp | pRtgRNA-NT-loxp, CrPAL-1 | This study |
| pRtgRNA-PAL-2-loxp | pRtgRNA-NT-loxp, CrPAL-2 | This study |
| pRtgRNA-2Spe2-loxp | pRtgRNA-Spe2-1-loxp, CrSpe2-2 | This study |
| pRtgRNA-2PAL-loxp | pRtgRNA-PAL-1-loxp, CrPAL-2 | This study |
| pRtEGT1 | pRtgRNA-NT-loxp, EGT1 expression cassette | This study |
| pRtMet14 | pRtgRNA-NT-loxp, Met14 expression cassette | This study |
| pRtSAM2 | pRtgRNA-NT-loxp, SAM2 expression cassette | This study |
| pRtSAH1 | pRtgRNA-NT-loxp, SAH1 expression cassette | This study |
| pRtADO1 | pRtgRNA-NT-loxp, ADO1 expression cassette | This study |
| pRtEGT1-EGT2 | pRtEGT1, RtEGT2 expression cassette | This study |
| pCAMBIA1301 | pVS1, *Kan^R^*, *Hyg^R^* | miaolingbio |
| pCM-Egt1 | pCAMBIA1301, RtEGT1 | This study |
| pCM-Egt2 | pCAMBIA1301, RtEGT2 | This study |
| pRtEGT1-2 | pRtgRNA-NT-loxp, New RtEGT1 expression cassette | This study |
| pRtEGT1-EGT2-2 | pRtEGT1-2, New RtEGT2 expression cassette | This study |
| pCM-Egt1-p2A2-Egt2 | pCM-Egt1, p2A2-Egt2 fragment | This study |
| pRtEGT1-p2A2-EGT2 | pRtgRNA-NT-loxp, New RtEGT1-p2A2-RtEGT2 expression cassette | This study |
| pRtEGT1-p2A1-EGT2 | pRtgRNA-NT-loxp, New RtEGT1-p2A1-RtEGT2 expression cassette | This study |
| pRtMet14-p2A1-SAM2-p2A1-SAH1 | pRtgRNA-NT-loxp, New Met14-p2A1-SAM2-p2A1-SAH1 | This study |
| pRtSAM2-p2A1-SAH1 | pRtgRNA-NT-loxp, New SAM2-p2A1-SAH1 | This study |

**Table S3**

| **Genes** | **20 nt spacer** | **sgRNA numbers** | **Positive rates** | **Mutation types** |
| --- | --- | --- | --- | --- |
| *Ku70* | AGGTTAGCGAGACAAGACTG | 1 | 0/4 | No |
| *Ku70* | GCTGTACGAGACGATCCATG | 1 | 1/4 | 15 nt deletion |
| *Ku70* | AGGTTAGCGAGACAAGACTG/ GCTGTACGAGACGATCCATG | 2 | 0/5 | No |
| *SPE2* | CTGCGTTCCCGACTCCCCGG | 1 | 0/4 | No |
| *SPE2* | AAGGTGTTGAGTGTGATCGA | 1 | 2/4 | 1 nt insertion; 4 nt deletion |
| *SPE2* | CTGCGTTCCCGACTCCCCGG/ AAGGTGTTGAGTGTGATCGA | 2 | 0/5 | No |
| *PAL* | GAACGTCTCCAAGATCTACG | 1 | 1/4 | 1 nt insertion |
| *PAL* | CTGGTCGATGAGCGAGACGA | 1 | 0/4 | No |
| *PAL* | GAACGTCTCCAAGATCTACG/ CTGGTCGATGAGCGAGACGA | 2 | 1/5 | 1 nt insertion; 438 nt deletion |

Nt represents nucleotide. No represents no mutation events detected.

**Table S4**

| **Genes** | **Homologs** | **Sequence identity** | **References** |
| --- | --- | --- | --- |
| RHTO_02079 (*Met14*) | YKL001C | 43.67% | [1] |
| RHTO_07080 (*SAM2*) | YDR502C | 66.67% | [1] |
| RHTO_01695 (*SAH1*) | YER043C | 64.86% | [2] |
| RHTO_05920 (*ADO1*) | YJR105W | 35.96% | [2] |
| RHTO_05506 (*SPE2*) | YOL052C | 33.65% | [1] |

Plasmid Construction. The plasmid pRtgRNA-Ku70-1-loxp was constructed as follows: The linear fragment was amplified from plasmid pRtgRNA-NT-loxp by using the primers crKu70-1-F/crKu70-1-R, and self-ligated to generate the plasmid pRtgRNA-Ku70-1-loxp. The plasmid pRtgRNA-Ku70-2-loxp was constructed as follows: The linear fragment was amplified from plasmid pRtgRNA-NT-loxp by using the primers crKu70-2-F/crKu70-2-R, and self-ligated to generate the plasmid pRtgRNA-Ku70-2-loxp. The plasmid pRtgRNA-2Ku70-loxp was constructed as follows: The sgRNA-Ku-2 expression cassette was amplified from plasmid pRtgRNA-Ku70-2-loxp by using the primers 2crKu-F/2crKu-R, and then ligated with the linear plasmid pRtgRNA-Ku70-1-loxp (digested by *Kpn*I), generating the plasmid pRtgRNA-2Ku70-loxp. The plasmids pRtgRNA-Spe2-1-loxp, pRtgRNA-Spe2-2-loxp, pRtgRNA-PAL-1-loxp, pRtgRNA-PAL-2-loxp were constructed via the similar steps of plasmid pRtgRNA-Ku70-1-loxp and pRtgRNA-Ku70-2-loxp, except for using the primers crSpe2-1-F/crSpe2-1-R, crSpe2-2-F/crSpe2-2-R, crPAL-1-F/crPAL-1-R, crPAL-2-F/crPAL-2-R to amplify the corresponding linear fragments, respectively. The plasmids pRtgRNA-2Spe2-loxp and pRtgRNA-2PAL-loxp were constructed via the similar steps of plasmid pRtgRNA-2Ku70-loxp, except for using the primers 2crSpe2-F/2crSpe2-R and 2crPAL-F/2crPAL-R to amplify the corresponding sgRNA-Spe2-2 and sgRNA-PAL-2 expression, and using the corresponding linear plasmid pRtgRNA-Spe2-1-loxp and pRtgRNA-PAL-1-loxp.

The plasmid pRtEGT1 was constructed as follows: The promoter *Ppgk* fragment was amplified from *Rhodotorula toruloides* 2.1389 by using the primers Ppgk-F/Ppgk-R and ligated with the linear plasmid pUC57 (digested by *EcoR*I), generating the plasmid pUC57-Ppgk. Then, the *RtEGT1* fragment (RHTO_05467) was amplified from *R. toruloides* 2.1389 by using the primers Egt1-F/Egt1-R and ligated with the linear plasmid pUC57-Ppgk (amplified by using the primers Egt1-GF/Egt1-GR), generating the plasmid pUC57-Ppgk-Egt1. Then, the terminator *Tnos* fragment was amplified from *R. toruloides* 2.1389 by using the primers NOS-F/NOS-R and ligated with the linear plasmid pUC57-Ppgk-Egt1 (digested by *Xba*I), generating the plasmid pUC57-Ppgk-Egt1-Tnos. Finally, the RtEGT1 expression cassette was amplified from the plasmid pUC57-Ppgk-Egt1-Tnos by using the primers PGK-xbaI-F/PGK-xbaI-R and ligated with the linear plasmid pRtgRNA-NT-loxp (digested by *Xba*I), generating the plasmid pRtEGT1. The plasmids pRtMet14, pRtSAM2, pRtSAH1, pRtADO1 were constructed via the similar steps of plasmid pRtEGT1, except for using the primers Met14-F/Met14-R, SAM2-F/SAM2-R, SAH1-F/SAH1-R, ADO1-F/ADO1-R to amplify the corresponding *Met14* (RHTO_02079), *SAM2* (RHTO_07080), *SAH1* (RHTO_01695), *ADO1* (RHTO_05920) fragments, and using the primers Met14-GF/Met14-GR, SAM2-GF/SAM2-GR, SAH1-GF/SAH1-GR, ADO1-GF/ADO1-GR to amplify the corresponding plasmid fragments (template: pUC57-Ppgk-Egt1-Tnos).

The plasmid pRtEGT1-EGT2 was constructed as follows: The promoter *Pgpd* fragment was amplified from *Rhodotorula toruloides* 2.1389 by using the primers Pgpd-F/Pgpd-R and ligated with the linear plasmid pUC57 (digested by *BamH*I), generating the plasmid pUC57-Pgpd. Then, the *RtEGT2* fragment (RHTO_04309) was amplified from *R. toruloides* 2.1389 by using the primers Egt2-F/Egt2-R and ligated with the linear plasmid pUC57-Pgpd (digested by *BamH*I), generating the plasmid pUC57-Pgpd-Egt2. Then, the terminator *Thsp* fragment was amplified from *R. toruloides* 2.1389 by using the primers hsp-F/hsp-R and ligated with the linear plasmid pUC57-Pgpd-Egt2 (digested by *Hind*III), generating the plasmid pUC57-Pgpd-Egt2-Thsp. Finally, the RtEGT2 expression cassette was amplified from the plasmid pUC57-Pgpd-Egt2-Thsp by using the primers GPD-xbaI-F/GPD-xbaI-R and ligated with the linear plasmid pRtEGT1 (digested by *Xba*I), generating the plasmid pRtEGT1-EGT2.

The plasmid pRtEGT1-EGT2-2 was constructed as follows: the *RtEGT1* fragment was amplified from *R. toruloides* 2.1389 by using the primers 1301-E1-F/1301-E1-R and ligated with the linear plasmid pCAMBIA1301 (digested by *Bgl*II and *BstE*II), generating the plasmid pCM-Egt1. The plasmids pCM-Egt2 was constructed via the similar steps of plasmid pCM-Egt1, except for using the primers 1301-E2-F/1301-E2-R. Then, the new RtEGT1 expression cassette was amplified from the plasmid pCM-Egt1 by using the primers NEGT1-F/NEGT2-R and ligated with the linear plasmid pRtgRNA-NT-loxp (digested by *Xba*I), generating the plasmid pRtEGT1-2. Finally, the RtEGT2 expression cassette was introduced to generate the plasmid pRtEGT1-EGT2-2 by the similar steps, except for using the primers NEGT2-F/NEGT2-R, and the corresponding plasmid pRtEGT1-2 fragments (digested by *Xba*I).

The plasmid pRtEGT1-p2A2-EGT2 was constructed as follows: The *RtEGT2* fragment, carrying p2A2 sequence (5’-GGAAGCGGAGCTACTAACTTCAGCCTGC

TGAAGCAGGCTGGAGACGTGGAGGAGAACCCTGGACCT-3’) derived from *Zaire ebolavirus*, was amplified by using the primers for the first round P2A1-F1/P2A1-R (template: pCM-Egt2) and the primers for the second round P2A1-F2/P2A1-R (template: the first round of PCR product), and then ligated with the linear plasmid pCM-Egt1 (digested by *BstE*II), generating the plasmid pCM-Egt1-ZP2A-Egt2. Finally, the RtEgt1-ZP2A-RtEgt2 expression cassette was amplified from the plasmid pCM-Egt1-ZP2A-Egt2 by using the primers NEGT1-F/NEGT2-R and ligated with the linear plasmid pRtgRNA-NT-loxp (digested by *Xba*I), generating the plasmid pRtEGT1-p2A2-EGT2. The plasmid pRtEGT1-p2A1-EGT2 was constructed via the similar steps of plasmid pRtEGT1-p2A2-EGT2, except for using another p2A1 sequence (5’-GGCTCGGGCGCTACCAACTTCTCGCTCCTCAAGCAGGCTGGCGACGTCGAAGAGAACCCTGGCCCT-3’) derived from *Porcine teschovirus*, and using two other pairs of primers P2A2-F1/P2A1-R and P2A2-F2/P2A1-R.

The plasmid pRtMet14-p2A1-SAM2-p2A1-SAH1 was constructed as follows: The *SAM2* fragment, carrying p2A1 sequence, was amplified from *R. toruloides* 2.1389 by using the primers MET14-SAM2-F1/SAM2-R and ligated with the linear plasmid pUC57-Ppgk-Met14-Tnos (amplified by using the primers MET14-SAM2-GF/MET14-SAM2-GR), generating the plasmid pUC57-Ppgk-Met14-SAM2-Tnos. Then, the *SAH1* fragment, carrying p2A1 sequence, was amplified from *R. toruloides* 2.1389 by using the primers 14-2-SAH1-F1/SAH1-R and ligated with the linear plasmid pUC57-Ppgk-Met14-SAM2-Tnos (amplified by using the primersSAH1-GF/14-2-SAH1-GR), generating the plasmid pUC57-Ppgk-Met14-SAM2-SAH1-Tnos. Finally, the entire Met14-p2A1-SAM2-p2A1-SAH1 expression cassette was amplified from the plasmid pUC57-Ppgk-Met14-SAM2-SAH1-Tnos by using the primers PGK-xbaI-F/PGK-xbaI-R and ligated with the linear plasmid pRtgRNA-NT-loxp (digested by *Xba*I), generating the plasmid pRtMet14-p2A1-SAM2-p2A1-SAH1. The plasmid pRtSAM2-p2A1-SAH1 was constructed via the similar steps of plasmid pRtMet14-p2A1-SAM2-p2A1-SAH1, except for using the template pUC57-Ppgk-SAM2-Tnos.

**References**

1. van der Hoek SA, Rusnák M, Wang G, Stanchev LD, de Fátima Alves L, Jessop-Fabre MM, Paramasivan K, Jacobsen IH, Sonnenschein N, Martínez JL, Darbani B, Kell DB, Borodina I. Engineering precursor supply for the high-level production of ergothioneine in *Saccharomyces cerevisiae*. Metab Eng. 2022;70:129-42.

2. Chen R, Gao J, Yu W, Chen X, Zhai X, Chen Y, Zhang L, Zhou YJ. Engineering cofactor supply and recycling to drive phenolic acid biosynthesis in yeast. Nat Chem Biol. 2022;18(5):520-29.
